# The HUSH complex controls brain architecture and protocadherin fidelity

**DOI:** 10.1126/sciadv.abo7247

**Published:** 2022-11-04

**Authors:** Astrid Hagelkruys, Marion Horrer, Jasmin Taubenschmid-Stowers, Anoop Kavirayani, Maria Novatchkova, Michael Orthofer, Tsung-Pin Pai, Domagoj Cikes, Sergei Zhuk, Meritxell Balmaña, Christopher Esk, Rubina Koglgruber, Paul Moeseneder, Jelena Lazovic, Lydia M. Zopf, Shane J.F. Cronin, Ulrich Elling, Jürgen A. Knoblich, Josef M. Penninger

**Affiliations:** ^1^ Institute of Molecular Biotechnology of the Austrian Academy of Sciences (IMBA), Vienna, Austria.; ^2^ Vienna Biocenter Core Facilities (VBCF), Vienna, Austria.; ^3^ Medical University of Vienna, Vienna, Austria.; ^4^ Department of Medical Genetics, Life Sciences Institute, University of British Columbia, Vancouver, Canada.

## Abstract

The HUSH (human silencing hub) complex contains the H3K9me3 binding protein M-phase phosphoprotein 8 (MPP8) and recruits the histone methyltransferase SETDB1 as well as Microrchidia CW-type zinc finger protein 2 (MORC2). Functional and mechanistic studies of the HUSH complex have hitherto been centered around SETDB1 while the in vivo functions of MPP8 and MORC2 remain elusive. Here, we show that genetic inactivation of *Mphosph8* or *Morc2a* in the nervous system of mice leads to increased brain size, altered brain architecture, and behavioral changes. Mechanistically, in both mouse brains and human cerebral organoids, MPP8 and MORC2 suppress the repetitive-like protocadherin gene cluster in an H3K9me3-dependent manner. Our data identify MPP8 and MORC2, previously linked to silencing of repetitive elements via the HUSH complex, as key epigenetic regulators of protocadherin expression in the nervous system and thereby brain development and neuronal individuality in mice and humans.

## Teaser

The HUSH complex plays important roles beyond transposable element regulation.

## INTRODUCTION

Histone methylation plays a crucial role in heterochromatin formation and is controlled by the opposing actions of “writers” (histone methyltransferases) and “erasers” (histone demethylases) (*1*, *2*). “Reader” proteins bind to certain histone modifications and thereby influence fundamental biological processes such as transcription, replication, or DNA repair. These epigenetic writers, erasers, and readers need to be tightly regulated, and disturbances in this highly balanced network of epigenetic control and chromatin-modifying enzymes can contribute to a variety of diseases including many types of cancers (*3*). Histone methylation has also been functionally linked to memory formation, neuroplasticity, and cognitive capabilities, and aberrant histone methylation has been associated with neurodevelopmental, neurodegenerative, and behavioral disorders (*4*–*6*).

To identify factors involved in the repression of silenced elements, several genome-wide screens have been performed in human cell lines and identified the HUSH (human silencing hub) complex (*7*–*9*). This recently discovered repressor complex, which contains M-phase phosphoprotein 8 (MPP8) and recruits the histone methyltransferase SETDB1 and Microrchidia CW-type zinc finger protein 2 (MORC2), has been implicated in silencing of genes, repetitive elements, and transgenes in mammals (*7*, *8*). So far, functional and mechanistic studies have focused on SETDB1 (*10*–*14*), while the in vivo functions of other HUSH complex members remain elusive. Recently, MORC2 mutations have been reported in individuals with Charcot-Marie-Tooth (CMT) disease type 2Z, a form of axonal neuropathy with progressive muscle weakness, atrophy, and sensory impairment (*15*–*17*) and in a neurodevelopmental disorder with intellectual disability, growth retardation, microcephaly, and variable craniofacial dysmorphism (*18*).

Here, we report that murine MPP8 and MORC2A are highly expressed in the brain, wherein they are exclusively found in neurons. Genetic inactivation of *Mphosph8* (coding for MPP8) or *Morc2a* in the nervous system of mice results in altered brain architecture, impaired motor functions, and reduced life span. Mechanistically, MPP8 and MORC2A precisely and selectively suppress the repetitive-like protocadherin gene cluster on mouse chromosome 18 in an H3K9me3-dependent manner, thereby affecting synapse formation. Moreover, we demonstrate that individual MPHOSPH8- or MORC2-deficient neurons in human cerebral organoids express increased numbers of clustered protocadherin isoforms. Our data uncover a novel role for the HUSH complex in the regulation of clustered protocadherins within the nervous system, thereby controlling brain development and neuronal fidelity in mice and humans.

## RESULTS

### Murine MPP8 and MORC2A are required for embryonic development

To elucidate the in vivo roles of MPP8 and MORC2A, we established mouse models carrying conditional *Mphosph8* or *Morc2a* knockout alleles. Exons 4 of *Mphosph8* and *Morc2a* were flanked by loxP sites (fig. S1, A and B) and Cre recombinase–mediated removal of this exon leads to out-of-frame splicing, subjecting the resulting mRNA to nonsense-mediated decay. Southern blot analysis of embryonic stem (ES) cells (fig. S1, C and D) identified correctly targeted clones, which were then used for blastocyst injections. Loss of *Mphosph8* or *Morc2a* in mouse ES cells resulted in no apparent changes in proliferation but were significantly impaired in their ability to form teratomas (fig. S1, E and F). *Actin*-Cre–mediated full-body knockout of *Mphosph8* or *Morc2a* in the mouse resulted in embryonic lethality (fig. S1, G and H). In comparison to a recent study showing that *Mphosph8* knockout mice display partial embryonic lethality with surviving *Mphosph8*^−/−^ mice developing normally apart from reduced body weight (*19*), in our study, *Actin*-Cre–mediated loss of *Mphosph8* resulted in 100% embryonic lethality before embryonic day 11.5 (E11.5) and loss of *Morc2a* led to embryonic lethality around E13.5 (fig. S1, I and J). These data show that MPP8 and MORC2A are critically required for teratoma formation and embryonic development.

### Deletion of *Mphosph8* or *Morc2a* in the nervous system results in elevated brain/body ratios and altered brain architecture

**Fig. 1.**
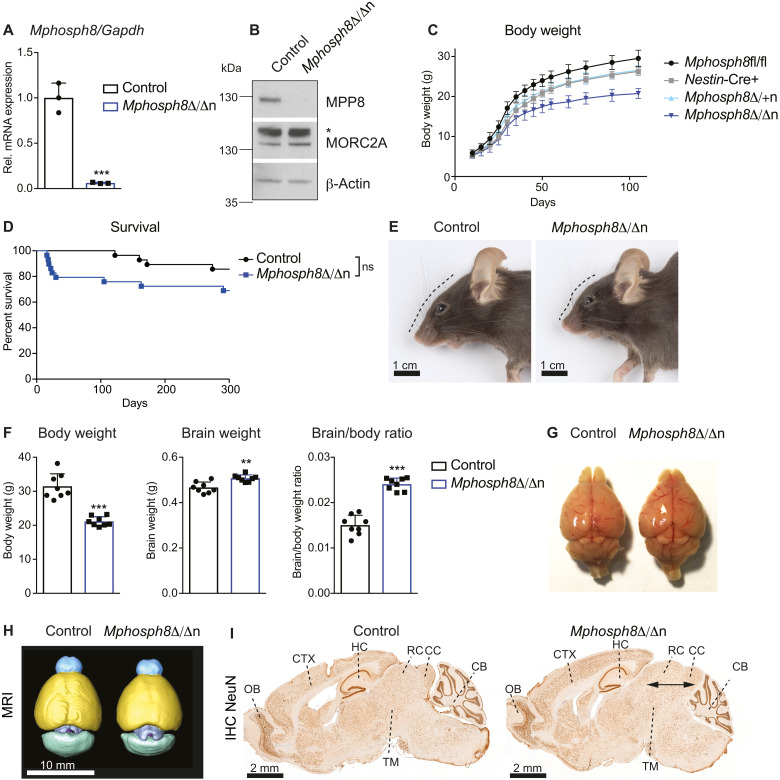
Altered brain architecture in *Mphosph8* knockout mice. (**A**) Relative mRNA expression of *Mphosph8* in littermate control and homozygous *Mphosph8*^Δ/Δn^ brains. Mean values ± SD were normalized to the housekeeping gene *Gapdh* (*n* = 3). Data are shown for one of two independent experiments. (**B**) Immunoblot analysis of littermate control versus *Mphosph8*^Δ/Δn^ whole-brain extracts using antibodies against MPP8, MORC2A, and β-actin as loading control. The asterisk indicates an unspecific band. (**C**) Body weights of *Mphosph8*^fl/fl^ (*n* = 18), *Nestin-*Cre+ (*n* = 8), heterozygous *Mphosph8*^Δ/+n^ (*n* = 7), and *Mphosph8*^Δ/Δn^ (*n* = 9) male littermates ± SD during the first 3 months of age. Data from several litters were pooled. (**D**) Kaplan-Meier survival curve over the first 300 days of control versus *Mphosph8*^Δ/Δn^ littermates. Data from several litters were pooled (*n* > 27), and survival curves were compared by the Log-rank (Mantel-Cox) test. (**E**) Representative macroscopic photographs of control (left) and *Mphosph8*^Δ/Δn^ (right) mouse heads (scale bars, 1 cm). (**F**) Body weights, brain weights, and brain/body ratios of control compared to *Mphosph8*^Δ/Δn^ adult (at 3 to 4 months) littermates. Data from two independent experiments were pooled and shown as mean values ± SD (*n* = 8). (**G** to **I**) Representative macroscopic images (G), in vivo MRI scans (H; scale bar, 10 mm), and NeuN immunohistochemistry (I; scale bars, 2 mm) of control (left) and *Mphosph8*^Δ/Δn^ (right) littermate adult (at 3 to 4 months) brains. In total, at least three mice per genotype were analyzed. OB, olfactory bulb; CTX, cerebral cortex; HC, hippocampus; RC, rostral colliculus; CC, caudal colliculus; TM, tegmentum; CB, cerebellum. Arrows indicate widening of collicular regions of the midbrain. For (A) and (F), each data point represents an individual mouse. *P* values were calculated using the Student’s *t* test. **P < 0.01; ***P < 0.001; ns, not significant.

**Fig. 2.**
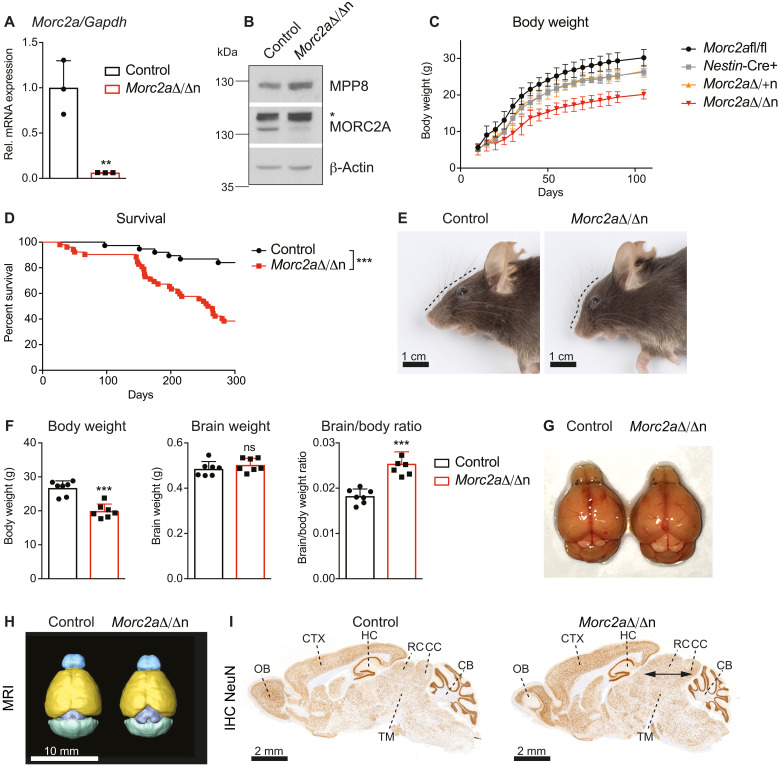
Altered brain architecture in *Morc2a* knockout mice. (**A**) Relative mRNA expression of *Morc2a* in littermate control and homozygous *Morc2a*^Δ/Δn^ brains. Mean values ± SD were normalized to the housekeeping gene *Gapdh* (*n* = 3). Data are shown for one of two independent experiments. (**B**) Immunoblot analysis of littermate control versus *Morc2a*^Δ/Δn^ whole-brain extracts with antibodies against MPP8, MORC2A, and β-actin as loading control. The asterisk indicates an unspecific band. (**C**) Body weights of *Morc2a*^fl/fl^ (*n* = 13), *Nestin-*Cre+ (*n* = 8), heterozygous *Morc2a*^Δ/+n^ (*n* = 8), and *Morc2a*^Δ/Δn^ (*n* = 11) male littermates (± SD) during the first 3 months of age. Data from several litters were pooled. (**D**) Kaplan-Meier survival curve over the first 300 days of control versus *Morc2a*^Δ/Δn^ littermates. Data from several litters were pooled (*n* > 35) and survival curves were compared by the Log-rank (Mantel-Cox) test. (**E**) Representative macroscopic photographs of control (left) and *Morc2a*^Δ/Δn^ (right) mouse heads (scale bars, 1 cm). (**F**) Body weights, brain weights, and brain/body ratios of control compared to *Morc2a*^Δ/Δn^ adult (at 3 to 4 months) littermates. Data from two independent experiments were pooled and are shown as mean values ± SD (*n* = 7). (**G** to **I**) Representative macroscopic images (G), in vivo MRI scans (H; scale bar, 10 mm), and NeuN immunohistochemistry (I; scale bars, 2 mm) of control (left) and *Morc2a*^Δ/Δn^ (right) littermate adult (at 3 to 4 months) brains. In total, at least three mice per genotype were analyzed. Arrows indicate widening of collicular regions of the midbrain. For (A) and (F), each data point represents an individual mouse. *P* values were calculated using the Student’s *t* test. **P < 0.01; ***P < 0.001.

On the basis of high expression of both MPP8 and MORC2A proteins in the adult mouse brain (fig. S1, K and L) and the known link between histone methylation, neuroplasticity, and cognitive abilities (*4*, *5*), we focused on investigating their function in the nervous system. Therefore, we crossed homozygous floxed *Mphosph8* and *Morc2a* mice to *Nestin*-Cre–expressing mice, effectively deleting *Mphosph8* or *Morc2a* in neuronal progenitors and postmitotic neurons from E11.0 onward. *Mphosph8*^fl/fl^ *Nestin*-Cre and *Morc2a*^fl/fl^ *Nestin*-Cre mice are hereafter referred to as *Mphosph8*^Δ/Δn^ and *Morc2a*^Δ/Δn^, respectively. Loss of *Mphosph8* and *Morc2a* was verified by quantitative reverse transcription polymerase chain reactions (qRT-PCRs; Figs. 1A and 2A) and immunoblots (Figs. 1B and 2B) from whole brains of control *Mphosph8*^fl/fl^ or *Morc2a*^fl/fl^ and their respective *Mphosph8*^Δ/Δn^ or *Morc2a*^Δ/Δn^ littermates. *Nestin*-Cre–mediated *Mphosph8* or *Morc2a* deficiency in the central nervous system resulted in reduced body sizes, body weights, and survival (Figs. 1, C and D, and 2, C and D).

Morphologically, both *Mphosph8*^Δ/Δn^ and *Morc2a*^Δ/Δn^ mice featured varying degrees of cranial alterations including skulls that ranged from normal to moderately domed (Figs. 1E and 2E). Nervous system–specific deletions of *Mphosph8* or *Morc2a* led to markedly elevated brain/body ratios (Figs. 1F and 2F). In both *Mphosph8*^Δ/Δn^ and *Morc2a*^Δ/Δn^ mice, the increase in brain mass corresponded to a partial macroencephaly featuring enlargement (dorsoventral and mediolateral expansion), predominantly of the collicular (tectal) and tegmental regions of the midbrain. This resulted in widening of the distance between the occipital lobes and the cerebellar vermis and imparted the appearance of exposed rostral and caudal colliculi. These changes were consistently observed by macroscopic examination (Figs. 1G and 2G) and magnetic resonance imaging (MRI; Figs. 1H and 2H), as well as in histologic sections (Figs. 1I and 2I). Quantitative immunohistochemistry revealed an increased density of RBFOX3^+^ (NeuN^+^) nuclei, especially in the colliculi (fig. S2, A to D), indicative of increased numbers of neurons. Glial fibrillary acidic protein immunohistochemistry did not reveal any apparent differences in astrocyte numbers and distribution, suggesting that the observed morphologic expansion was attributable to increases in the neuronal rather than astrocytic components of the neuropil (fig. S2, E and H). Notably, we found no histomorphological alterations in the olfactory bulb, cerebellar folia, the cerebral cortex, or the hippocampal formation (fig. S2, C, D, F, G, I, and J). These data show that neuronal loss of murine MPP8 or MORC2A results in a morphological and neuronal expansion of defined brain areas.

### *Mphosph8* and *Morc2a* double mutant mice

To study whether the loss of both MPHOSPH8 and MORC2A leads to more severe phenotypes, we generated *Mphosph8*^Δ/Δn^ *Morc2a*^Δ/Δn^ mice lacking both *Mphosph8* and *Morc2a* in the nervous system. Loss of *Mphosph8* and *Morc2a* was verified by quantitative RT-PCRs and immunoblots from whole brains of control (*Mphosph8*^fl/fl^ *Morc2a*^fl/fl^) and *Mphosph8*^Δ/Δn^ *Morc2a*^Δ/Δn^ littermates (fig. S3, A and B). Upon *Nestin-*Cre–mediated double deletion of both *Mphosph8* and *Morc2a*, these mice showed elevated brain/body ratios and altered brain architecture including exposed colliculi, very similar to the changes observed in *Mphosph8*^Δ/Δn^ or *Morc2a*^Δ/Δn^ single-mutant mice (fig. S3, C to H). We observed a synergistic effect on mortality whereby survival rates of double-mutant mice decreased after already 150 days to 50% and after 300 days to 20% (fig. S3I). These data indicate that, apart from enhanced lethality, double knockout of *Mphosph8* and *Morc2a* leads to similar brain architecture phenotypes as in single-mutant mice, suggesting that these two proteins act in the same molecular complex.

### *Mphosph8* or *Morc2a* deficiency affects motor and memory functions

**Fig. 3.**
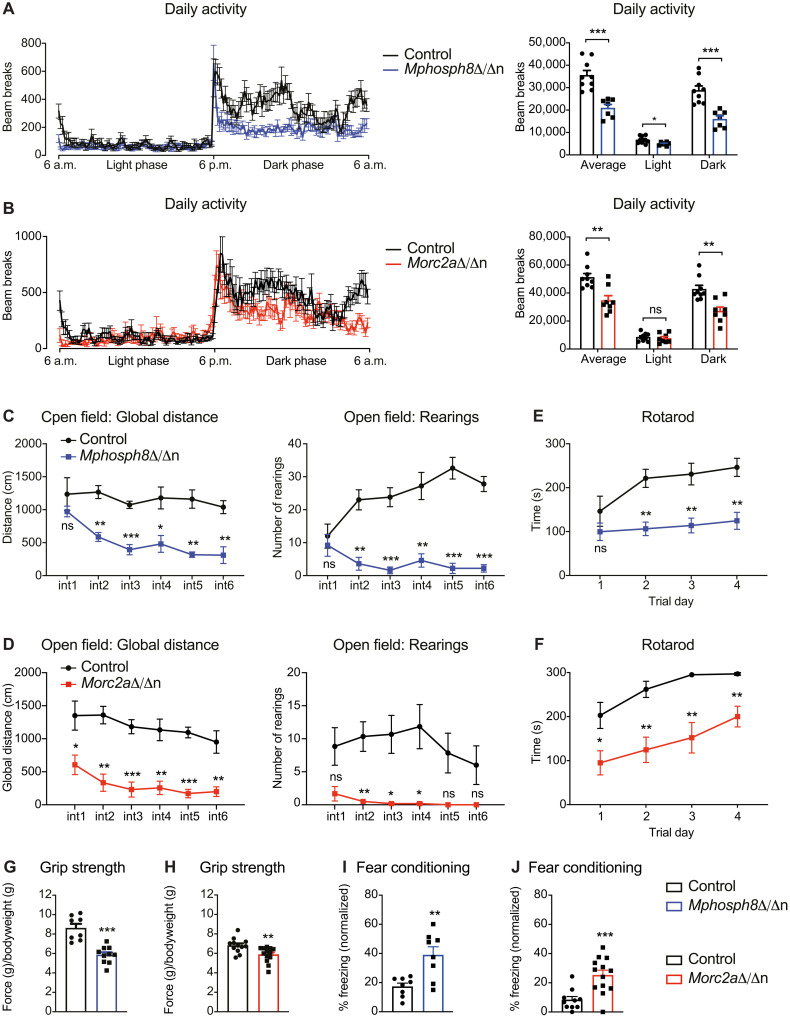
Decreased motor performance and enhanced fear memory upon loss of *Mphosph8* or *Morc2a*. (**A** and **B**) Daily activity in PhenoMaster cages measured by beam breaks of *Mphosph8*^Δ/Δn^ mice (A, *n* > 5) and *Morc2a*^Δ/Δn^ mice (B, *n* > 7) compared to their respective control littermates over 24 hours. Data from two independent experiments were pooled and shown as mean values ± SEM. (**C** and **D**) Global distance and number of rearings of *Mphosph8*^Δ/Δn^ mice (C) and *Morc2a*^Δ/Δn^ mice (D) compared to their control littermates in the open field test. Data are shown as mean values ± SEM (*n* > 5) and are representative of one of two independent experiments. (**E** and **F**) Latency to fall from the accelerating Rotarod of *Mphosph8*^Δ/Δn^ mice (E) and *Morc2a*^Δ/Δn^ mice (F) compared to control littermates. Data from two independent experiments were pooled and shown as mean values ± SEM (*n* > 8). (**G** and **H**) Four-paw grip strength normalized to body weights of *Mphosph8*^Δ/Δn^ mice (G) and *Morc2a*^Δ/Δn^ mice (H) compared to control littermates. Data from two independent experiments were pooled and shown as mean values ± SEM (*n* > 7). (**I** and **J**) Percent freezing in the fear-context box normalized to training without foot shock of *Mphosph8*^Δ/Δn^ mice (I) and *Morc2a*^Δ/Δn^ mice (J) compared to control littermates. Data from two independent experiments were pooled and shown as mean values ± SEM (*n* > 7). For the right panels in (A) and (B) and for (G) to (J), each data point represents an individual mouse. *P* values were calculated using the Student’s *t* test. *P < 0.05; **P < 0.01; ***P < 0.001.

To evaluate the pathophysiological consequences of *Mphosph8* or *Morc2a* neuronal inactivation, we performed various behavioral tests analyzing locomotion, learning, and memory in adult mice. Since *Mphosph8*^Δ/Δn^ and *Morc2a*^Δ/Δn^ mice showed the most notable neuroanatomic alterations in the midbrain, we paid special attention to motor control and coordination abilities. Both *Mphosph8* and *Morc2a* single-knockout mice exhibited decreased locomotor activity in PhenoMaster cages (Fig. 3, A and B) and in the open field test (Fig. 3, C and D). Moreover, the mutant mice exhibited reduced motor performance and impaired motor functions on the Rotarod test (Fig. 3, E and F). To exclude *Nestin*-Cre–specific effects, we performed similar experiments comparing *Nestin*-Cre^−^ and *Nestin*-Cre^+^ mice and observed no significant differences in any of these assays (fig. S4, A to C). *Mphosph8*^Δ/Δn^ and *Morc2a*^Δ/Δn^ mice also exhibited reduced neuromuscular function in the grip strength test (Fig. 3, G and H), similar to the findings in human patients with *MORC2* mutations (*18*).

*Mphosph8*^Δ/Δn^ and *Morc2a*^Δ/Δn^ mice did not show any changes in the spontaneous alternation performance in the Y-maze as well as anxiety in the elevated plus maze, but both knockout mice showed improved hippocampal-dependent fear-context memory (Fig. 3, I and J, and fig. S4, D to G). While the mice showed no apparent differences in finding the visual platform in the Morris water maze, *Mphosph8*^Δ/Δn^ and, to some extent, *Morc2a*^Δ/Δn^ mice performed worse in finding the hidden platform in the Morris water maze test in short-term and long-term probe trials, indicating impaired spatial learning (fig. S4, H and I). *Mphosph8* and *Morc2a* knockout mice did not show any evidence of ataxia or gait-related problems using the catwalk assay (fig. S4, J and K). Thus, increased midbrain sizes and accompanying brain architectural changes in *Mphosph8* and *Morc2a* mutant mice are associated with defective motor functions and spatial learning, yet improved fear-context memory.

To noninvasively determine physiological and functional changes in the mouse brain at rest, we used resting-state functional MRI (fMRI). The level of oxygen supply is synchronized throughout the brain, and this synchrony can be measured as amplitude of low-frequency fluctuation (ALFF) of the signal (*20*). Different stressors and neurological disturbances can lead to disruption of the synchrony and manifest as changes in ALFF signal. Both *Mphosph8*^Δ/Δn^ and *Morc2a*^Δ/Δn^ mice showed changes in resting-state activation with significantly increased neuronal activation throughout the cortex and reduced activation in the posterior brain (fig. S5, A and B). The similar patterns of brain activation found in both *Mphosph8* and *Morc2a* knockout mice suggest that MPP8 and MORC2A play similar roles in neuronal activation and synaptic connectivity. Together, this indicates that deletion of *Mphosph8* and *Morc2a* results in comparable alterations of defined brain circuits at rest.

### Up-regulation of clustered protocadherins upon loss of *Mphosph8* or *Morc2a*

Since MPP8 is part of the repressive HUSH complex, we assessed transcriptional changes upon loss of *Mphosph8* and performed RNA sequencing (RNA-seq) from control *Nestin*-Cre^+^ and *Mphosph8*^Δ/Δn^ littermate whole brains at postnatal day 14. To estimate repetitive element enrichment using the RNA-seq data, we used the *DESeq2* and *piPipes* pipelines and detected no major changes in repetitive elements, with the exception of a slight up-regulation of major satellite repeats (GSAT_MM) and some LTRs and LINE elements (fig. S6, A to C). Differentially expressed protein coding genes contained only two down-regulated (including *Mphosph8*) and 55 up-regulated genes (fold change >2, adjusted *P* value <0.05), which were highly enriched in synapse assembly, synapse organization, and cell-cell adhesion gene ontology categories (fig. S6D). Twenty-one of the fifty-five up-regulated genes were from the protocadherin (Pcdh) gene cluster on chromosome 18 (fig. S6E). These clustered protocadherins comprise a large family of cell surface molecules expressed in the developing vertebrate nervous system. In contrast to classic cadherin genes, many of the encoded ectodomains of each member of the protocadherin gene cluster are present in one large exon and do not contain any introns (*21*). Besides clustered protocadherins, 11 other genes consisting of only one exon were significantly up-regulated more than twofold (fig. S6E). This is in accordance with a recent study showing that the HUSH complex represses long intronless elements including LINE-1 retrotransposons and intronless transgenes, which are thereby recognized as invading genetic elements (*22*) and suggests that MPP8 and MORC2A silence clustered Pcdhs because of their intronless repetitive-like nature. Pcdh combinations in individual neurons provide a barcode that mediates the precise molecular recognition of synaptic partners and are involved in synapse development and neuronal fidelity (*23*–*25*). This family of adhesion molecules has also been implicated in brain evolution (*26*, *27*).

**Fig. 4.**
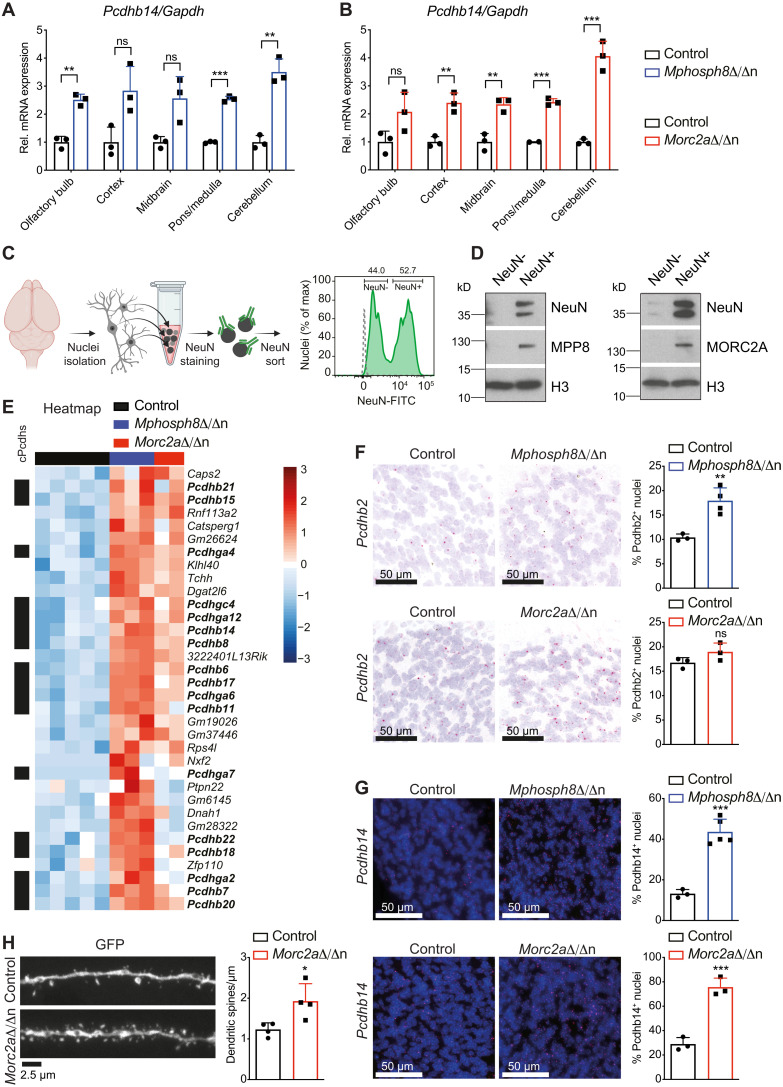
Loss of murine *Mphosph8* or *Morc2a* leads to up-regulation of clustered protocadherins and increased synaptic spines. (**A** and **B**) Relative mRNA expression of *Pcdhb14* in littermate control versus *Mphosph8*^Δ/Δn^ (A) and control versus *Morc2a*^Δ/Δn^ (B) brain areas including the olfactory bulb, cortex, midbrain, pons/medulla, and cerebellum. Mean values ± SD were normalized to the housekeeping gene *Gapdh* (*n* = 3) and the control values were set to 1. (**C**) Schematic illustration (created with Biorender.com) and representative plot of flow cytometry–based sorting of NeuN-immunotagged brain nuclei. The gray dashed line indicates unstained nuclei as background control. (**D**) Immunoblot analysis of NeuN^−^ and NeuN^+^ wild-type brain nuclear extracts as depicted in (C) using antibodies against MPP8, MORC2A, and histone H3 (H3) as loading control. (**E**) Heatmap of up-regulated genes (adjusted *P* value < 0.05, fold change >2 between control and *Mphosph8*^Δ/Δn^) found in an RNA-seq experiment using sorted brain NeuN^+^ nuclei from control, *Mphosph8*^Δ/Δn^, and *Morc2a*^Δ/Δn^ adult littermates. *Z* score is shown from −3 (blue) to 3 (red). (**F** and **G**) In situ hybridization (RNAscope) for *Pcdhb2* (F) and *Pcdhb14* (G) mRNA and quantification in the whole brain of adult (at 3 to 4 months) control versus *Mphosph8*^Δ/Δn^ (top, *n* > 2) and control versus *Morc2a*^Δ/Δn^ (bottom, *n* = 3) littermates (scale bars, 50 μm). A representative image of the granular cell layer of the cerebellum is shown. Data are shown as mean values ± SD. (**H**) Representative maximum-intensity-projection and quantification of z-stacked confocal images of hippocampal CA1 pyramidal cell distal dendrites in control versus *Morc2a*^Δ/Δn^ (*n* = 4; per mouse, three to six individual neurons were counted and averaged) adult littermates (scale bar, 2.5 μm). Data are shown as mean values ± SD. For (A) and (B) and (F) to (H), each data point represents an individual mouse. *P* values were calculated using the Student’s *t* test. *P < 0.05; **P < 0.01; ***P < 0.001.

The up-regulation of protocadherins was detected comparably two- to fourfold in different brain regions upon loss of both *Mphosph8* or *Morc2a* as representatively shown for *Pcdhb14* (Fig. 4, A and B). Because of the neuron-specific expression of protocadherins, we optimized a protocol for flow cytometry–based sorting of NeuN-immunotagged brain nuclei. Fluorescence-activated cell sorting (FACS) of freshly isolated neuronal nuclei (NeuN^+^) and non-neuronal nuclei of the brain (NeuN^−^) revealed an almost exclusive expression of MPP8 and MORC2A in neurons but not in non-neuronal cells of the brain (Fig. 4, C and D, and fig. S7A). Loss of either *Mphosph8* or *Morc2a* resulted in an up-regulation of clustered protocadherins (cPcdhs) in NeuN^+^ neuronal nuclei (Fig. 4E and fig. S7B). The observed up-regulation of cPcdhs could stem from the fact that the expression level of already expressed protocadherins is elevated or that there are increased numbers of protocadherins per cell, i.e., more cells express any particular cPcdh. To explore these hypotheses, we analyzed the expression of two specific protocadherins—*Pcdhb2* and *Pcdhb14*—by chromogenic RNA in situ hybridization (RNAscope) in *Mphosph8*^Δ/Δn^ and *Morc2a*^Δ/Δn^ brains. We indeed found a higher number and proportion of *Pcdhb2*- and *Pcdhb14*-positive cells compared to control littermates (Fig. 4, F and G). To test whether elevated cPcdh expression might expand the neuronal barcodes and increase synaptic connections, we crossed *Morc2a*^Δ/Δn^ animals to mice expressing enhanced green fluorescent protein (EGFP) under the control of a neuronal promoter [Tg(Thy1-EGFP).MJrs mice], which allowed direct examination of dendritic spines in vivo (*28*). We indeed observed increased density of spines in *Morc2a*^Δ/Δn^ brains (Fig. 4H). These data show that in *Mphosph8*^Δ/Δn^ and *Morc2a*^Δ/Δn^ brains, individual neurons express more clustered protocadherin isoforms and exhibit a higher number of synaptic connections.

### *Mphosph8*- and *Morc2a*-deficient neurons show decreased H3K9 trimethylation at the protocadherin cluster

**Fig. 5.**
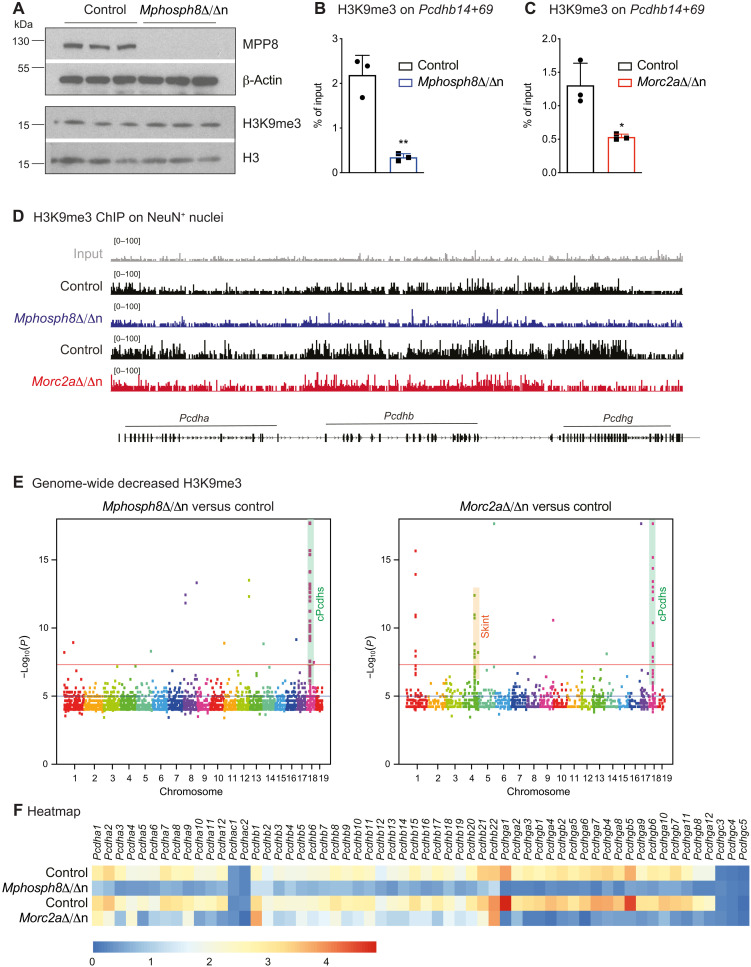
Specifically, the protocadherin cluster shows less H3K9 trimethylation in *Mphosph8*-deficient or *Morc2a*-deficient neurons. (**A**) Immunoblot analysis of littermate control versus *Mphosph8*^Δ/Δn^ brain extracts with antibodies against MPP8 and H3K9me3 as well as β-actin and total histone H3 as loading controls. (**B** and **C**) Chromatin from control versus *Mphosph8*^Δ/Δn^ littermate (B, *n* = 3) and control versus *Morc2a*^Δ/Δn^ littermate (C, *n* = 3) NeuN^+^ nuclei was immunoprecipitated with an H3K9me3-specific antibody followed by qPCR with primers specific for *Pcdhb14* (+69 bp from the transcriptional start site). Data are representative of one of two independent experiments and are shown as mean values ± SD. (**D**) Normalized read density plots of H3K9me3 ChIP-seq in brain NeuN^+^ nuclei from control, *Mphosph8*^Δ/Δn^, and *Morc2a*^Δ/Δn^ mice for the protocadherin cluster on chromosome 18. (**E**) Manhattan plot visualization of H3K9me3 ChIP-seq differential analysis using diffreps in 1-kb sliding windows showing localized enrichments for decreased abundance of H3K9me3 methylation in NeuN^+^ nuclei of *Mphosph8*^Δ/Δn^ versus control mice (left, *n* = 3) and *Morc2a*^Δ/Δn^ versus control mice (right, *n* = 2). All the top-scoring dots on chromosome 18 correspond to the clustered *Pcdh* locus (highlighted in green). For *Morc2a*^Δ/Δn^, all the top-scoring dots on chromosome 4 correspond to the *Skint* gene cluster (highlighted in orange). (**F**) Heatmap of the normalized H3K9me3 intensity of H3K9me3 ChIP-seq in brain NeuN^+^ nuclei from *Mphosph8*^Δ/Δn^ and *Morc2a*^Δ/Δn^ mice compared to their respective littermate controls. For control versus *Mphosph8*^Δ/Δn^ nuclei, data from three individual mice were pooled. To compare control versus *Morc2a*^Δ/Δn^ nuclei, data from two individual mice were pooled. RPKM are shown from 0 (blue) to 5 (red). For (B) and (C), each data point represents an individual mouse. *P* values were calculated using the Student’s *t* test. *P < 0.05; **P < 0.01.

To establish neuronal individuality and generate functional neuronal circuits, each neuron transcribes only a few Pcdh isoforms from the protocadherin gene cluster differentially and independently, while the silent protocadherins are marked by DNA methylation and H3K9me3 to maintain a repressed state (*29*–*31*). To investigate whether the observed up-regulation of cPcdhs was associated with decreased trimethylation of H3K9, we first assessed global H3K9me3 levels in *Mphosph8*^Δ/Δn^ brains and did not detect any differences (Fig. 5A). When analyzing freshly isolated adult control, *Mphosph8*^Δ/Δn^, and *Morc2a*^Δ/Δn^ NeuN^+^ neuronal nuclei by chromatin immunoprecipitation (ChIP) experiments for abundance of the histone methylation mark H3K9me3, we, however, detected markedly decreased H3K9me3 levels at the *Pcdhb14* locus (Fig. 5, B and C) despite the limited cell numbers that could be obtained and posed challenges for this experiment. In additional, independently performed H3K9me3 ChIP-seq experiments, chromosome 18 with the clustered protocadherins emerged as the top-scoring locus for H3K9me3 hypomethylation in both *Mphosph8*^Δ/Δn^ and *Morc2a*^Δ/Δn^ neuronal nuclei as visualized in the genome browser views (Fig. 5D), genome-wide Manhattan plots (Fig. 5E), and H3K9me3 intensity heatmaps (Fig. 5F). Notably, individual biological replicates for the analysis of differential genome-wide H3K9me3 abundancy were merged to overcome the low signal-to-noise ratio and the low sequencing depth of the ChIP-seq experiment (Fig. 5, E and F). When compared to differentially H3K9me3-methylated regions in *Setdb1* knockout versus wild-type cortical neurons (*14*), we found similar patterns (fig. S7C). In *Morc2a*^Δ/Δn^ mice, another hotspot of H3K9me3 hypomethylation was the *Skint* gene cluster on chromosome 4 (Fig. 5E and fig. S7D), which encodes proteins with immunoglobulin (Ig)–like domains thereby playing a role in direct cell-cell signaling in the immune system (*32*). Together, these data indicate that *Mphosph8* or *Morc2a* deficiency in neurons leads to decreased histone H3K9 trimethylation specifically on the protocadherin cluster, resulting in up-regulation of cPcdhs.

### Human cerebral organoids lacking *MPHOSPH8* or *MORC2* express increased numbers of clustered protocadherins at the single-cell level

To translate our mouse data and to model the role of MPHOSPH8 and MORC2 in human brain development, we generated human three-dimensional (3D) cerebral organoids (*33*, *34*). We established two independent clones of *MPHOSPH8* and *MORC2* knockout H9 human ES cell lines by CRISPR-Cas9 engineering and used these clones for cerebral organoid generation. We observed an up-regulation of MORC2 upon loss of MPHOSPH8 and vice versa, suggesting a potential compensation in the mutant ES cell lines (fig. S8A), which, to some extent, was also detected in *Mphosph8*^Δ/Δn^ and *Morc2a*^Δ/Δn^ mouse brains (Figs. 1B and 2B). We used these ES cell lines to generate cerebral organoids as previously described (*34*). Single-cell RNA-seq analysis of day 27 cerebral organoids confirmed the enrichment of neuronal progenitors (fig. S8, B and C). Fluorescent immunohistochemistry of day 60 cerebral organoids further revealed the presence of both neuronal progenitors and neurons (fig. S8D). *MPHOSPH8* and *MORC2* knockout human H9 ES cells and cerebral organoids showed a strong up-regulation of repetitive elements including LINE-1 and alpha satellites (fig. S9, A, B, and D), demonstrating that the HUSH complex regulates expression of defined transposons in human stem cells and developing cerebral organoids.

**Fig. 6.**
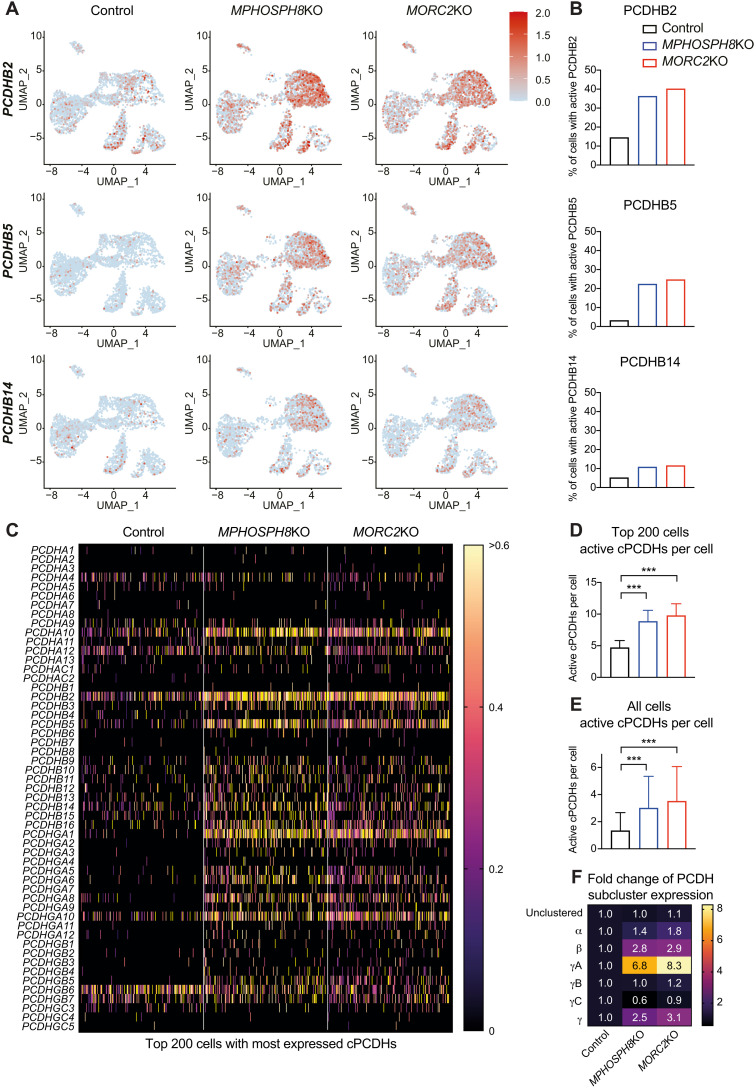
MPHOSPH8 and MORC2 control fidelity of clustered protocadherins in human cerebral organoids. (**A**) Expression of *PCDHB2* (top), *PCDHB5* (middle), and *PCDHB14* (bottom) in control, *MPHOSPH8*KO, and *MORC2*KO single cells from day 27 cerebral organoids projected on a uniform manifold approximation and projection plot. Each dot represents one cell, and in total, approximately 3400 cells per group were analyzed. (**B**) Percent of cells expressing *PCDHB2* (top), *PCDHB5* (middle), and *PCDHB14* (bottom) in control (*n* = 3488), *MPHOSPH8*KO (*n* = 3414), and *MORC2*KO (*n* = 3426) single cells from day 27 cerebral organoids as shown in (A). (**C**) Heatmap of the normalized expression of the top 200 cells with the most expressed cPCDHs in control, *MPHOSPH8*KO, and *MORC2*KO single cells from day 27 cerebral organoids, shown for all clustered protocadherins. Log-normalized UMI counts are shown from 0 (black) to >0.6 (yellow). (**D**) Number of expressed clustered protocadherins per single cell of the top 200 cells with the most expressed cPCDHs in control, *MPHOSPH8*KO, and *MORC2*KO single cells from day 27 cerebral organoids as shown in (C). Mean values ± SD are shown. (**E**) Number of expressed clustered protocadherins per single cell in control (*n* = 3488), *MPHOSPH8*KO (*n* = 3414), and *MORC2*KO (*n* = 3426) single cells from day 27 cerebral organoids. Mean values ± SD are shown. (**F**) Relative fold increase of expressed unclustered and clustered protocadherins per single cell in *MPHOSPH8*KO and *MORC2*KO single cells from day 27 cerebral organoids in comparison to the control, shown for each subcluster separately. For (A) to (F), for each group (control, *MPHOSPH8*KO, and *MORC2*KO), two organoids (each organoid originating from an independent ESC clone) were pooled. For (D) and (E), *P* values were calculated using ordinary one-way analysis of variance using multiple comparisons. ***P < 0.001.

While clustered protocadherins are expressed at a very low level in pluripotent human ES cells, an up-regulation of clustered protocadherins could be observed in *MPHOSPH8*- or *MORC2*-deficient cerebral organoids at different time points of differentiation as exemplified by *PCDHB2* (fig. S9C). Single-cell RNA-seq of day 27 cerebral organoids revealed an increased proportion of cells expressing a defined protocadherin upon loss of *MPHOSPH8* or *MORC2* (Fig 6, A and B). The higher number of cells expressing a particular protocadherin was observed throughout all cell clusters including neuronal progenitors (expressing *SOX2*, *PAX6*, and *DLX1*) and immature neurons (expressing *DCX*; Fig. 6A and fig. S8C). Moreover, upon *MPHOSPH8* or *MORC2* knockout, individual cells expressed a higher number of clustered protocadherins (Fig. 6, C to E), especially in the *PCDHB* and *PCDHGA* subclusters (Fig. 6F). Thus, our human cerebral organoid data show that individual *MPHOSPH8*- or *MORC2*-deficient neurons express increased numbers of clustered protocadherins.

## DISCUSSION

In this study, we identify murine M-phase phosphoprotein 8 (MPP8) and Microrchidia CW-type zinc finger protein 2 (MORC2A) as crucial regulators of brain development and function. Full-body knockout of either *Mphosph8* or *Morc2a* leads to embryonic lethality and *Nestin-*Cre–mediated nervous system–specific loss of *Mphosph8* or *Morc2a* results in decreased survival and altered brain morphology with expansion of the midbrain leading to increased cerebro-cerebellar distance and more exposed rostral and caudal colliculi. The alterations in neuromorphology, neuronal activity, and behavior, including motor deficits and differences in learning and memory, are remarkably similar in *Mphosph8* and *Morc2a* knockout mice. Mechanistically, *Mphosph8* and *Morc2a* are exclusively expressed in neurons, where they repress the protocadherin cluster on mouse chromosome 18 in an H3K9me3-dependent manner, thereby affecting synapse formation.

The combinatorial expression of clustered protocadherins in individual neurons generates barcodes for neuronal identity as well as synapse formation and thereby provides the molecular basis for neuronal diversity, neuronal network complexity, and function of the vertebrate brain. In the mouse, the 58 clustered protocadherin genes are tandemly arrayed in α, β, and γ subclusters called *Pcdh*α*, Pcdh*β, and *Pcdh*γ, which encode 14, 22, and 22 cadherin-like proteins, respectively, with each having its own promoter (*35*, *36*). The expression of different protocadherin combinations from each of the three gene clusters in individual neurons provides barcodes to distinguish self from nonself and ensures that neurons only interact with other neurons, while they do not contact processes of the same neuron with the same barcode (*37*, *38*). By RNAscope RNA in situ hybridization and single-cell RNA-seq, we observed that individual MPHOSPH8- or MORC2-deficient neurons in both mouse brains and human cerebral organoids express increased numbers of clustered protocadherin isoforms. ChIP data suggest that the up-regulation of cPcdhs is likely due to loss of HUSH-deposited H3K9me3 across clustered protocadherin genes. This is in accordance with a study showing that the histone methyltransferase SETDB1 regulates a large neuron-specific topological domain including the protocadherin cluster and loss of murine neuronal *Setdb1* triggers structural disintegration specifically of this megabase-scale topologically associated domain (*14*). However, because of the challenges in obtaining sufficiently high numbers of sorted neurons for our experiments, as well as the limitations in antibody specificity and resulting low signal-to-noise ratio, additional effects or other causes cannot be excluded. Together, this suggests that the HUSH-complex containing MPP8 and SETDB1 partners with the chromatin remodeler MORC2 to regulate the 3D chromatin structure of the protocadherin gene cluster in neuronal cells. Since SETDB1 does not remodel chromatin on its own, it remains to be investigated whether the structure across the clustered protocadherin genes is ultimately formed by MORC2. In our mutant mice, we also observed increased synapse formation and region-specific differences in neuronal activity measured by fMRI. It requires further elucidation how this is associated with the impaired motor functions, spatial learning deficits, and the improved fear-context memory observed in *Mphosph8*- or *Morc2a*-deficient mice.

Some of the observed mouse phenotypes including growth retardation, craniofacial dysmorphisms, and weakness are similar to human patients with *MORC2* mutations (*18*), while others are opposite (microcephaly versus macrocephaly), which might stem from the fact that patients carry heterozygous *MORC2* mutations in the adenosine triphosphatase (ATPase) module resulting in apparent hyperactivation of HUSH silencing. A heterozygous full-body p.S87L *Morc2a* mouse mutant (mimicking one of the various hyperactivating patient mutations) showed phenotypes similar to our *Morc2a*-deficient mice concerning locomotive dysfunction and activity, but featured neuronal apoptosis, smaller cerebella, cerebellar ataxia, and spinal cord motor neuron degeneration (*39*). It remains to be investigated whether the p.S87L *Morc2a* mice also exhibit deregulation of clustered protocadherins. Different knockout mouse models of protocadherins in defined tissues cause massive neuronal loss and a smaller spinal cord area (*40*) or a thinned retina (*23*). The opposing phenotypes of *Morc2a*^Δ/Δn^ and p.S87L *Morc2a* mutant mice in regard to brain size and numbers of neurons might, therefore, be due to p.S87L *MORC2A* being a better HUSH effector with more stable functional ATPase dimers (*41*), ultimately exerting opposite effects on expression of the protocadherin cluster. Since dysregulation of clustered protocadherins is associated with a variety of neurological and neurodevelopmental diseases as well as mental disorders including autism spectrum disorder, bipolar disorder, Alzheimer’s disease, cognitive impairments, and schizophrenia (*42*, *43*), our data on the key importance of the HUSH complex in protocadherin gene expression might provide new understanding on the epigenetic regulation of such diseases.

In addition to the up-regulation of clustered protocadherins, we observed a strong derepression of alpha satellite repeats in human cerebral organoids and a moderate up-regulation of major satellite repeats in our knockout mice. These abundant tandem repeats are located at the site of centromere attachment and in the neighboring pericentromeric regions and are known to be bound by heterochromatin protein 1 (HP1) (*44*). The mammalian chromodomain-containing methyl-H3K9 reader protein MPP8 and chromo-like domain–containing MORC2 do not have orthologs either in *Drosophila* or in *Caenorhabditis*. This suggests that they could function through different mechanisms compared to the conserved chromodomain-containing reader protein HP1 and hence represent a second evolutionary route to H3K9me3-mediated heterochromatin regulation in mammalian cells. The up-regulated clustered protocadherins are mostly vertebrate-specific and might mediate neurite self-avoidance by specifying single-cell identity similar to invertebrate Dscam1 proteins. Clustered protocadherins can also be found in some invertebrates such as coleoid cephalopods including octopuses, which have the largest nervous systems among invertebrates and a rich behavioral repertoire including complex problem solving, observational learning, and a sophisticated adaptive coloration system (*45*). The octopus genome encodes 168 protocadherin genes, nearly three-quarters of which are found in tandem clusters on the genome (*26*). Thus, both octopuses and vertebrates have independently evolved a diverse array of clustered protocadherin genes. The synaptic specificity regulated by members of the protocadherin gene cluster thus allows the enormous increase in number and complexity of neuronal subtypes, synaptic connections, and the neural networks of the vertebrate brain compared to most of its invertebrate ancestors. Whether a HUSH-like complex universally controls protocadherin barcoding and regulates brain and cognitive evolution remains to be determined.

Fine-tuning of neural connectivity is important for advanced cerebral functions as well as brain and cognitive evolution. Our data now show that the HUSH complex, previously linked to silencing of transposons, has a role in regulating sizes and proportions of brain regions and synapse formation through epigenetic regulation of protocadherin expression.

## MATERIALS AND METHODS

### Mice

For *Mphosph8*, the ES cell clone EPD0058_2_C01 (Mphosph8^tm1a(EUCOMM)Wtsi^) was purchased from EUCOMM. For *Morc2a*, the targeting vector PG00072_X_1_H08 (KOMP) was electroporated into IB10/C ES cells generating the Morc2a^tm1a(KOMP)Wtsi^ allele. In both cases, exon 4 was flanked by loxP sites and the adjacent LacZ-Neo selection cassette was flanked by Frt sites. Southern blot analysis of ES cells identified correctly targeted clones, which were then used for blastocyst injections to create chimeric mice. These mice were bred on a C57BL/6J genetic background, the LacZ-Neo cassette was removed by crossing mice to an *Actin*-FlpE deleter line [B6.Cg-Tg(ACTFLPe)9205Dym/J], and after various breeding steps, homozygous floxed alleles were generated. Then, homozygous floxed *Mphosph8* and *Morc2a* mice were crossed to either *Actin*-Cre [Tmem163^Tg(ACTB-cre)2Mrt^] or *Nestin*-Cre [B6.Cg-Tg(Nes-cre)1Kln/J] mice (*46*, *47*). To express EGFP under control of the neuronal *Thy1* promoter and thereby label neurons, mice were crossed to Tg(Thy1-EGFP)MJrs mice (*28*). Mouse genotypes were assessed by PCR (see table S1 for genotyping primer sequences). Notably, only age- and sex-matched littermates from respective crosses were used for experiments. Female and male mice were included in the trials, with no sex differences noted for any test. When not explicitly stated, mouse experiments were carried out 10 to 16 weeks after birth. All mice were bred, maintained, examined, and euthanized in accordance with institutional animal care guidelines and ethical animal license protocols approved by the legal authorities. All experimental animal projects were approved by the Federal Ministry of Education, Science, and Research. Experiments were performed in accordance with the ARRIVE guidelines. Dorsal and lateral photographs of the head were acquired from selected littermates of each genotype with a Nikon D7 digital camera and a Nikon AF-S Micro Nikkor 105 mm 1:2:8 G VR lens.

### Behavioral experiments

Behavior experiments were carried out under the animal license number GZ:2020-0.392.948 according to Austrian legislation. The open field, Y-maze, elevated plus maze, fear conditioning, grip strength, Morris water maze, and CatWalk tests were performed by the Preclinical Phenotyping Facility at the Vienna Biocenter Core Facilities GmbH (VBCF), Vienna Biocenter (VBC), Austria. For testing, mice were transferred to the preclinical phenotyping facility of the VBCF at least 1 week before experiments and housed at a 14-hour light/10-hour dark cycle in IVC racks with access to food and water ad libitum. Before each experiment, mice were allowed to habituate to the experimental room for at least 30 min before any testing.

#### Open field test

Naïve mice were allowed to explore an open field arena (www.tse-systems.com) sized 50 cm (width) by 50 cm (length) by 29.5 cm (height) for 30 min with the release from the center and video-tracked using TSE VideoMot 3D version 7.01 software. In the software, a “center” zone was defined as a central square 25 cm by 25 cm in size, the rest being the “border zone.” Light conditions were 200 lux in the center zone. The time spent in each zone, distances traveled, numbers of center visits, and rearings were recorded as readout parameters.

#### Y-maze test

The Y-maze was performed as a test for working memory using a custom-built Y-shaped maze with gray, opaque walls and floor with the following dimensions: arm length, 30 cm; arm width, 6 cm; and wall height, 14.5 cm. After at least 30 min of habituation to the test room (180 lux, visual cues on walls), mice were placed individually into the end of one of the three arms (arms A, B, and C), facing the wall at the end of the arm, and allowed to explore the maze for 5 min while being video-tracked using the Topscan software (Cleversys Inc., USA). The experimenter watched the videos in the same room, behind a curtain and scored the latency to leave the starting arm (which was alternated between the mice) and the numbers and sequences of arm entries. These sequences were evaluated in terms of the triplet arms. Three arm entries in a row were scored as either correct spontaneous alternations (SA; e.g., BAC, CBA, and ABC), erroneous alternate arm returns (AARs; e.g., BAB, CBC, and ABA), or erroneous same arm returns (SARs, e.g., BAA, CCB, and AAC). After each triplet was scored, the start of the analysis was shifted by one entry and the next triplet sequence was scored. Two such shifts of analyses result in overlapping triplets and the scoring of all possible decision points. Spontaneous alternation performance was calculated as [spontaneous alternations (SA)/(total arm entries – 2)].

#### Elevated plus maze test

Mice were placed in the center zone (6.5 by 6.5 cm), facing an open arm of a custom-built elevated plus maze (elevated 54 cm above the floor) with two open arms (OA, 30 cm length, 7 cm width) and two wall-enclosed arms (closed arms, CA, 30 cm length, 6 cm width, walls 14.5 cm high). Exploration paths were video-tracked for 5 min using the Topscan software (Cleversys Inc., VA, USA), and the amount of time spent and distances traveled in the open arms, closed arms, and center zone were recorded. Lux levels were 180 lux in the center zone and open arms and 35 lux in the closed arms.

#### Grip strength test

Grip strength was measured using a grip strength meter (Bioseb, USA). For forelimb measurements, the mouse was gently lowered over the top of a grid so that only its front paws could grip the grid. The animal was gently pulled back, and when it released the grid, which is connected to a sensor, the maximal grip strength value of the animal was displayed on the screen and noted. For the forelimb and hindlimb measurements, the mouse was gently lowered over the top of the grid so that both its front and hind paws could grip the grid. The torso was kept parallel to the grid and the mouse was gently pulled back steadily until the grip was released down the complete length of the grid. The maximal grip strength value of the animal was recorded. Both “forelimb only” and “fore- and hindlimb” tests were performed in an alternating fashion three times per mouse with 15-min intertrial intervals, and the values were averaged among the three trials.

#### Morris water maze

Mice were trained to swim in a pool (diameter: 1 m) with colored water (white paint used: OBI PU Buntlack schneeweiß glänzend 17.05.23) treated with aquarium cleaner (TetraAqua aquarium conditioner, according to the manufacturer’s instructions) at a temperature of 20° to 22°C. The platform (diameter: 10 cm) was submerged about 8 mm under the water surface, using the visual cues placed in the room for orientation. Mice were placed in the water facing the wall of the pool and given two sessions a day with four trials per session using alternating entry points in different quadrants for each trial and video-tracked using the software Topscan 3.0 (Cleversys Inc., VA, USA). On day 1, the visual capacity of the mice was assessed by making the platform visible with a black flag and letting the mice explore the pool for 1 min or until they found the platform. If they did not find the platform within 1 min of the first session, they were guided toward the platform by pointing a pair of forceps in front of their nose. After a break on days 2 and 3, mice were given hidden platform training from days 4 to 8 without any marking of the platform. The time needed to find the platform was recorded. After the last trial on day 8, mice were tested for short-term memory by removing the platform and letting the mice explore the pool for 1 min. This probe trial was repeated in the morning of day 11 to test for long-term memory. The time spent searching in the target quadrant and target zone (exact location of the platform) was evaluated.

#### Fear conditioning

In the morning of day 1 (9:00 a.m. to 1:00 p.m.) mice were trained to associate the conditioned sound stimulus (CS = 85 dB, 10 kHz) to an unconditioned foot shock stimulus (US = 0.5 mA) using the following protocol: 120-s prephase (no sound, no shock), 30-s CS with the last 2 s coupled to the US, 90-s intertone interval (no sound, no shock), 30-s CS with the last 2 s coupled to the US, and 70-s postphase (no sound, no shock) in a Coulbourn Habitest operant cage (Coulbourn Instruments, MA, USA, www.coulbourn.com) using house light in the visible range. Mice were video-recorded using the software FreezeFrame from Actimetrics, IL, USA (www.actimetrics.com). For context testing, mice were placed back in the same conditioning box 24 hours later and observed for 4 min without any sound or shock presentation. For the cue test, mice were placed in the same fear conditioning box, but with changed appearance (different texture of walls and floor, infrared house light, no ventilator, and lemon aroma spotted in box) for approximately 28 hours (in the afternoon session 1:00 p.m. to 5:00 p.m.) after the context test and the following protocol was applied: 120-s prephase (no sound, no shock), 60-s CS (only sound, no shock), 60-s intertone interval, 60-s CS (only sound, no shock), and 60-s postphase (no sound, no shock). Freezing was defined as a minimum of 2 s without movement except breathing and was analyzed using the FreezeView software from Actimetrics, IL, USA (www.actimetrics.com).

#### CatWalk test

Gait was analyzed using the CatWalk XT system (Noldus Information Technology) essentially as described in (*48*) with the following exceptions: Mice were not trained to reach a goal box but to directly reach the homecage. Training was performed on the first day and the test was done on two consecutive days directly after the training day. An average of three trials performed on each test day was calculated for each mouse and then an average across the two test days was calculated for each animal.

#### Motor activity in PhenoMaster cages

Activity measurements were performed at room temperature (21° to 23°C) on a 12-hour light/12-hour dark cycle in a PhenoMaster System (TSE systems, Bad Homburg, Germany) using an open-circuit calorimetry system and animals were checked daily by veterinary staff. Mice were housed individually, trained on drinking nozzles for 72 hours, and allowed to adapt to the PhenoMaster cage for 2 days. Food and water were provided ad libitum in the appropriate devices and measured by the built-in automated instruments. Activity parameters were measured for five consecutive days.

#### Accelerating Rotarod

Up to four mice were placed simultaneously on the rod compartments of a Rotarod device (Ugo Basile) to evaluate the motor ability. The assays were conducted in the afternoon, between 2:00 p.m. and 5:00 p.m. One day before the test sessions, the mice were trained to stay on the Rotarod apparatus with a rotation of 5 rpm for 5 min as a habituation trial. From days 1 to 4, the mice were assayed with accelerating mode from 5 to 40 rpm for 5-min time periods each. The latencies until the mice fell were recorded and passive rotations (clinging onto the rod without running) were treated like a fall.

### Magnetic resonance imaging

MR images were acquired in the Preclinical Imaging Facility at the Vienna Biocenter Core Facilities GmbH (VBCF), Vienna Biocenter (VBC), Austria, with a 15.2-T Biospec horizontal bore scanner (Bruker BioSpin) and a BFG6S-100 actively shielded gradient system (750 mT/m maximum gradient strength). A quadrature transmit/receive volume coil (23 mm inner diameter; Bruker BioSpin) was used. During imaging, all mice were anesthetized with 1.5% isoflurane. High-resolution 3D imaging was done using gradient echo sequence (TR = 160 ms, TE = 3.5 ms, voxel size 100 × 100 × 100 μm^3^, NEX = 3, total scan duration 1 hour 47 min). Each 3D image set was manually segmented using Amira-Avizo software version 2019.1 (Thermo Fisher Scientific, Waltham, MA, USA). The delineation of different brain structures was performed in the axial plane and subsequently controlled in the other two planes. A mouse brain atlas was used as a reference (*49*). The brain surface and structures were delineated on the basis of the MRI signal intensity differences.

### Resting-state fMRI

All animals were imaged using 15.2-T small-animal MRI (Bruker BioSpec) and 23-mm birdcage coil. Before imaging, all mice were anesthetized with 4% isoflurane (induction only) and then reduced to 1.5%. During imaging, care was taken to adjust the isoflurane levels immediately so that respiration did not fall below 140 breaths per minute (bpm) at any time. During imaging, respiration was maintained between 140 and 160 bpm. For the resting-state fMRI study, a single-shot spin echo EPI sequence with spin echo readout was used (TR = 2000 ms, TE = 19.7 ms, FOV = 16 × 16 mm^2^, voxel size = 250 × 250 μm^2^, 30 slices 0.5 mm thick, one average, 360 repetitions, 12-min total imaging time). Each time, following functional imaging, a high-resolution T1-weighted anatomical scan was acquired using gradient echo sequence (TR = 500 ms, TE = 3 ms, FOV = 16 × 16 mm^2^, voxel size = 125 × 125 μm^2^, 30 slices 0.5 mm thick, four averages).

### Teratoma formation

For teratoma formation, 1 million ES cells (Haplobank) were mixed with Matrigel (Corning, 356231) and injected subcutaneously into Crl:NU(NCr)-Foxn1<nu> nude mice. Teratoma growth was monitored for 4 to 6 weeks.

### Histology, immunostaining, and in situ hybridization

Mouse brains were harvested, macroscopically inspected, and fixed overnight by immersion in 4% paraformaldehyde, processed with an automated tissue processor (Logos, Milestone Medical or Donatello, DiaPath/Sanova), embedded in paraffin, and sectioned at a thickness of 2 μm with a standard rotary microtome (Microm HM 355, Thermo Fisher Scientific). Sections were stained with hematoxylin and eosin on an automated staining platform (HMS 740, Microm or Gemini AS, Thermo Fisher Scientific) or with Luxol Fast Blue–Cresyl Violet (LFBCV) using a standard manual protocol. Immunohistochemistry for the Neuronal Nuclear Antigen (NeuN) was performed using an automated immunostainer (Bond III, Leica or Infinity i6000, Biogenex). Slides were rehydrated, subjected to heat-induced antigen retrieval in a citrate buffer, and incubated with a mouse anti-NeuN antibody (clone A60, Millipore, MAB377; 1:100). The used secondary antibodies were mouse linker antibody (Abcam, ab133469) and a goat anti-rabbit antibody (Dako, E0432; 1:500). Chromogenic detection was performed with the Bond Intense R Detection System (Leica, DS9263) or the DCS Supervision 2 Polymer System (Innovative Diagnostik-Systeme, PD000POL-K) and the DAB substrate kit (Abcam, ab64238). In situ hybridization for Protocadherin beta 2 (*Pcdhb2*) was performed with a manual protocol using the RNAscope Probe - Mm-Pcdhb2 (ACD, 46781) followed by chromogenic detection using the RNAscope 2.5 HD Reagent Kit - RED (ACD). In situ hybridization for Protocadherin beta 14 (*Pcdhb14*) was performed using the RNAscope Probe - Mm-Pcdhb14 (ACD, 544981) followed by fluorescent detection with the RNAscope Multiplex Fluorescent Detection Kit v2 (ACD) and Opal 4-color flHC Kit (PerkinElmer).

Slides were reviewed by a board-certified pathologist with an Axioskop 2 MOT microscope (Zeiss). Whole-slide images were prepared using the Pannoramic FLASH 250 III whole slide scanner (3D Histech) with the 40×/0.95 plan apochromat objective and Adimec Quartz Q12A180 camera for bright-field and a pco.edge 4.2 4MP camera for fluorescent samples. Representative images were acquired from slides with a SPOT Insight 2 camera (Spot Imaging, Diagnostic Instruments) and from whole-slide images with the Case Viewer Software (3D Histech). Quantification of NeuN-positive cells was performed with QuPath, an open-source whole slide image analysis platform (*50*). For quantification of positive cells in the cortex, diencephalon, tectum (rostral and caudal colliculi), and tegmentum, these regions were manually annotated in each image within each workspace. For quantification of positive cells in the cerebellum, the granular layer and other regions (molecular layer and white matter) were separately delineated with the ANN_MLP Pixel classifier. Positive cells were detected by setting the intensity thresholds for the nuclear DAB OD mean score compartment. The threshold was applied to all images within the workspace with a command history script. Detection was performed with separate thresholds and scripts for (i) the granular layer of the cerebellum, (ii) the molecular and white matter layers of the cerebellum, and (iii) all the other indicated regions of the brain. Examples of the regions and corresponding cellular detections are presented in fig. S2 (C and D). Quantification of *Pcdhb2*- and *Pcdhb14*-positive spots was performed by automated quantification of nuclei and positive spots in the whole brain using nuclei detection via StarDist plugin (QuPath) and spot detection in QuPath with exported TIFF files from whole slide images.

### Synaptic number analysis

*Mphosph8*^Δ/Δn^ and *Morc2a*^Δ/Δn^ mice were crossed to Tg(Thy1-EGFP)MJrs mice (*28*) to express EGFP under control of the neuronal Thy1 promoter. Mice were perfused, brains were fixed overnight by immersion in 4% paraformaldehyde, cryo-protected in phosphate-buffered saline (PBS) with 30% sucrose, embedded in optimal cutting temperature compound (Sakura), and sagittally sectioned at a thickness of 10 μm. After counterstaining with 4′,6-diamidino-2-phenylindole (DAPI) and mounting in fluorescent mounting medium (Dako), the slides were analyzed using the LSM700 Axio Imager confocal microscope using a 63× objective. Synapse number counts were performed manually using FIJI software 2.1.0 (*51*).

### qRT-PCR and qRT-PCR analysis

Tissues and cells were isolated and homogenized in TRIzol reagent (Invitrogen). Total RNA was isolated according to the manufacturer’s instructions. RNA was reverse-transcribed with the iScript cDNA Synthesis Kit (Bio-Rad). Real-time PCR analysis was performed with GoTaq qPCR master mix (Promega) on a CFX384 system (Bio-Rad). Data were normalized to values for the housekeeping gene *Gapdh* for mouse samples and *ACTB* (encoding β-actin) for human samples. All primers used in this study are listed in table S1.

### mRNA sequencing and data analysis

cDNA libraries for each of the samples were generated from total RNA using the NEB PolyA Enrichment Kit following the manufacturer’s instructions. Fifty–base pair (bp) single-read (SR50) sequencing was performed on HiSeq 2500 at the VBCF NGS Unit (www.viennabiocenter.org/facilities) using v4 SBS reagents. RNA-seq reads were trimmed using trim-galore v0.5.0 and reads mapping to abundant sequences included in the iGenomes UCSC GRCm38 reference (mouse rDNA, mouse mitochondrial chromosome, phiX174 genome, adapter) were removed using bowtie2 v2.3.4.1 alignment. The remaining reads were aligned to the mouse genome (Ensembl GRCm38 release 94) using star v2.6.0c and reads in genes were counted with featureCounts (subread v1.6.2) and TEcount v2.0.3. Differential gene expression analysis on raw counts of genes or genes plus repeat elements was performed using DESeq2 v1.18.1, and gene set overrepresentation analysis was performed with clusterprofiler 3.6.0 in R v3.4.1. TEtoolkit, which uses multimapping read reassignment via an expectation-maximization (EM) algorithm and family-level quantification, was used to counteract analytical biases because of transposable element (TE) mapping ambiguity especially for recently active TE families (*52*). Nevertheless, due to the short-read sequencing strategy, younger TE families might be underrepresented or underestimated in the current analysis (*53*). The data discussed in this publication have been deposited in NCBI’s Gene Expression Omnibus (*54*) and are accessible through the GEO Series accession number GSE185330 (www.ncbi.nlm.nih.gov/geo/query/acc.cgi?acc=GSE185330).

### Protein isolation and immunoblotting

For protein extraction, cells or tissues were manually homogenized in Hunt buffer [20 mM tris-HCl (pH 8.0), 100 mM sodium chloride, 1 mM EDTA, and 0.5% NP-40] supplemented with Halt protease/phosphatase inhibitor cocktail (Thermo Fisher Scientific). After full-speed centrifugation, the supernatant containing the soluble protein fraction was further used. Equal amounts of 20 to 30 μg of protein were separated by SDS–polyacrylamide gel electrophoresis and transferred onto polyvinylidene difluoride membranes (Immobilion-P, Merck Millipore) according to standard protocols. Blots were blocked for 1 hour with 5% milk in TBST [1× tris-buffered saline (TBS) and 0.1% Tween 20] and were then incubated overnight at 4°C with primary antibodies diluted in 5% milk in TBST. Blots were washed three times in TBST for 5 min and further incubated with horseradish peroxidase–conjugated secondary anti-mouse–IgG–H&L chain (Promega) or anti-rabbit-IgG-F(ab′)2 (GE Healthcare) antibody for 1 hour at room temperature, washed three times in TBST for 5 min, and visualized using enhanced chemiluminescence (ECL, GE Healthcare). See table S1 for a list of antibodies used in this study. β-Actin was used to control for protein loading.

### Sorting of neuronal nuclei

Freshly isolated mouse brains were homogenized in nuclear isolation buffer (10 mM tris-HCl, 50 mM sodium disulfite, 1% Triton X-100, 10 mM MgCl_2_, and 8.6% sucrose, pH 6.5) and centrifuged for 5 min at 455*g* at 4°C. The resulting pellet was washed four times with 900 μl of nuclear isolation buffer with centrifugation at 455*g* at 4°C. The pelleted nuclei were then resuspended in 1.4 ml of PBS, filtered through a cell strainer, and stained with a 1:1000 dilution of the anti-NeuN antibody (clone A60, Alexa Fluor 488–conjugated, Millipore MAB377X) for 45 min by rotation in the dark at 4°C after adding bovine serum albumin (BSA) to a final concentration of 0.1%. After washing in PBS, the stained nuclei were filtered through FACS tubes, sorted on a FACS Aria III (BD Biosciences) sorter in PBS, pelleted 5 min at 590*g* at 4°C, and the pellet was immediately used for RNA isolation or frozen at −80°C.

### Chromatin immunoprecipitation

Freshly isolated brains were finely minced and homogenized in PBS, washed with PBS, and cross-linked by adding formaldehyde (to a final concentration of 1%) at room temperature for 10 min. The cross-linking process was stopped by the addition of glycine to a final concentration of 125 mM. The homogenized and cross-linked brains were washed in PBS and used for fluorescence-activated sorting of neuronal brain nuclei (NeuN^+^) as described above. After the sort, the pelleted nuclei were immediately resuspended in ChIP lysis buffer (50 mM tris-HCl, 10 mM EDTA, and 1% SDS, pH 8.1). The chromatin isolation procedure was followed as previously described (*55*). For ChIP assays, equal amounts of Bioruptor-sonicated chromatin were diluted 10-fold and precipitated overnight with the following antibodies: H3K9me3 (Abcam) and rabbit IgG (Invitrogen) as a control. Chromatin antibody complexes were isolated using protein A beads for rabbit primary antibodies or G-beads for mouse primary antibodies (Dynabeads, Invitrogen). The PCRs with 1:20 dilutions of genomic DNA (input) were carried out together with the precipitated DNA. The extracted DNA was used for quantitative PCR analysis using the primers listed in table S1 and also for ChIP-seq.

For ChIP-seq input, DNA was quantified using Quant-iT PicoGreen dsDNA Assay (Thermo Fisher Scientific). One nanogram of input DNA was used for library preparation with the Ultra Low Input Library Kit (Qiagen, 180497). The procedure of library preparation was done according to the manual provided by Qiagen with an adapter dilution of 1:100. Quality control of the libraries included a fragment analyzer run and qPCR to determine average size and precise concentration. On the basis of the qPCR values, samples were pooled equimolarly to a concentration of 2.5 nM. The pool was loaded on an Illumina HiSeq2000 flowcell with a concentration of 18 pM and sequenced 50-bp single end. ChIP-seq reads were trimmed using Trim Galore v0.6.4 and thereafter aligned to the mm10 reference genome (igenomes) using bowtie v2.4.1. The sorted BAM was filtered to remove reads in blacklisted regions using bedtools v2.29.2 and mm10.blacklist.bed.gz v1, and alignments with a MAPQ score below 10 using samtools v1.9. Duplicated reads were marked and excluded with Picard MarkDuplicates v2.23.3. Sequence alignments were converted to bed format using bedtools, and differential ChIP-seq sites for merged replicates were called using diffReps v1.55.6 with settings --meth gt --window 1000.

The total number of reads and the alignment rates were the following:

66590_input: 10,072,887 total reads with an alignment rate of 97.65

66591_WT_H3K9me3: 10,571,810 total reads with an alignment rate of 96.77

66595_WT_H3K9me3: 9,432,555 total reads with an alignment rate of 96.84

66608_WT_H3K9me3: 9,076,968 total reads with an alignment rate of 95.91

66592_Mphosph8KO_H3K9me3: 9,979,521 total reads with an alignment rate of 97.1

66596_Mphosph8KO_H3K9me3: 9,785,475 total reads with an alignment rate of 96.93

66609_Mphosph8KO_H3K9me3: 10,005,564 total reads with an alignment rate of 96.64

66611_Morc2aKO_H3K9me3: 8,810,357 total reads with an alignment rate of 95.86

66612_Morc2aKO_H3K9me3: 8,968,962 total reads with an alignment rate of 87.18

For a detailed view of the normalized ChIP-seq read coverage per Pcdh exon, we retrieved exon positions in the genomic region chr18:37863700–36843100 (mm10 refGene/UCSC), collapsed overlapping exons using bedtools merge (v2.27.1), and kept flattened regions assigned to a single *Pcdh* gene only. ChIP-seq reads within those remaining exonic segments were recorded using featureCounts (Rsubread v2.4.3) and the filtered sequence alignments as input. Counts were converted to RPKM (Reads Per Kilobase per Million mapped reads) before visualization.

### Deletion of MPHOSPH8 and MORC2 in human ES cells

The human ES cell (hESCs) line WA09 (H9) was obtained from WiCell, verified to display a normal karyotype. H9 cells were cultured feeder-free on hESC-qualified Matrigel (Corning, 354230)–coated plates in E8 medium (IMBA Stem Cell Core Facility) in Dulbecco’s modified Eagle’s medium (DMEM)/F-12 (Gibco, 11320033) with an Antibiotic-Antimycotic cocktail (Thermo Fisher Scientific, 15240062). All stem cells were maintained in a 5% CO_2_ incubator at 37°C. Cells were split using 0.5 mM UltraPure EDTA (Invitrogen, 15575-038) in PBS to wash once, then incubated for 3 min, EDTA aspirated, rinsed in E8 medium, and plated on Matrigel-coated plates. For the generation of knockout cells, the pSpCas9(BB)-2A-Puro (PX459: Addgene, 62988) plasmid was used carrying a guide RNA targeting *MPHOSPH8* (clone C7 and C11: gCATAGACGATCACAAAACCA) or *MORC2* (clone A6 and C4: gACCAAACAAGAATTCGTGAG). H9 cells were nucleofected using the Amaxa nucleofector (Lonza) with the P3 primary cell 4D nucleofector kit L (Lonza LONV4XP-3024). Puromycin (Invivogen; 10 mg/ml, ant-pr-1) selection with 0.2 μg/ml in E8 medium was started 2 days after transfection. Puromycin was removed after 2 days, and the cells were further kept in E8 medium. After selecting bulk clones by TIDE analysis (http://shinyapps.datacurators.nl/tide), single-cell colonies were picked and verified to carry frameshift mutations. Loss of MPHOSPH8 and MORC2 protein in hESCs was confirmed by immunoblots.

### Human cerebral organoids

Cerebral organoids were generated and cultured as previously described (*34*) with slight modifications. Briefly, H9 hESC cells were grown to 60 to 80% confluency, washed with PBS, and single-cell suspensions were obtained using Accutase (Sigma-Aldrich, A6964). Pelleted cells were resuspended in E8 medium supplemented with RevitaCell cell supplement (Invitrogen, A2644501) and counted. A total of 9000 cells were seeded per well to form embryoid bodies in a 96-well ultralow-attachment U-bottom plate (Sbio, MS-9096UZ) in 150 μl of E8 medium supplemented with RevitaCell. On day 3, medium was changed to E8, and from days 6 to 13, organoids were grown in neural induction media [∼1 liter of neural induction media containing 1 liter of DMEM/F-12, 10 ml of N2 supplement (IMBA Molecular Biology Service), 10 ml of GlutaMAX (Invitrogen, 35050-038), 10 ml of MEM-NEAA (Sigma-Aldrich, M7145), and 1 ml of heparin (Sigma-Aldrich, H3149) of a 1 mg/ml dilution], slightly different as previously described (*34*, *56*). On day 10, embryoid bodies were embedded into Matrigel (Corning, 354234) droplets, transferred to a 10-cm dish, and cultured from day 13 until day 25 in differentiation medium without vitamin A [∼1 liter of differentiation media containing 500 ml of DMEM/F-12 and 500 ml of neurobasal media (Invitrogen, 21103049), 5 ml of N2 supplement, 10 ml of GlutaMAX, 5 ml of MEM-NEAA, 20 ml of B27 supplement minus vitamin A (Invitrogen, 12587010), 350 μl of a 1:100 dilution of 2-mercaptoethanol (Merck, 8057400250), and 250 μl of insulin [Sigma-Aldrich, I9278-5ML)] and then in differentiation medium with vitamin A (IMBA Molecular Biology Service), 0.4 mM vitamin C (Sigma-Aldrich, 255564-100G), and 0.1% m/v NaHCO_3_ (Merck, 1.06329.1000). From day 20 onward, cerebral organoids were constantly shaken on a horizontal shaker.

### Single-cell RNA-seq of cerebral organoids

Single-cell suspensions from two cerebral organoids per condition were prepared at day 27 using 0.9×:1× Accutase/Trypsin (Gibco, 11538876) and incubated for 15 min on 37°C while shaking and dissociating manually by pipetting every 5 min. Digestion was stopped by gradually adding two volumes of cold DMEM/F-12, washed with DPBS^−/−^ (Thermo Fisher Scientific, 14190094) plus 0.04% BSA (VWR, 9048-46-8). Cells were then filtered through a cell strainer, counted, and loaded onto a 10x Genomics controller in DPBS^−/−^ plus 0.04% BSA according to the manufacturer’s protocol with a target cell number of 4000 cells. Sequencing was performed on an Illumina NextSeq2000 lane and analyzed using 10x Cell Ranger 4.0.0 workflow, reference refdata-gex-GRCh38-2020-A and Seurat 3.2.2.

### Immunohistochemistry of cerebral organoids

Organoids were washed three times in PBS and fixed overnight at 4°C in 4% paraformaldehyde. The next day, the organoids were washed again three times in PBS and dehydrated overnight at 4°C in 30% sucrose. The organoids were then embedded with OCT in a multiwell format and stored at −80°C until cryo-sectioning. The block was cut in 20-mm sections, and the sections were stored at −80°C. For immunostaining, the slides were dried at room temperature, washed three times in PBS, and blocked in 4% BSA and 0.5% Triton X-100 in PBS. Afterward, the sections were incubated with the primary antibody overnight in a humidified chamber. The next day, the sections were washed three times in washing solution (PBS containing 0.05% Triton X-100) and incubated for 2 hours at room temperature in the secondary antibody and DAPI (Invitrogen, D3571; dilution 1:1000). The slides were afterward washed three times in washing solution, mounted, and sealed. See table S1 for a list of primary and secondary antibodies used for immunostaining of the cerebral organoids.

### Statistical analysis

All values are given as means ± SDs unless stated otherwise (SEM in behavior experiments in Fig. 3 and fig. S4). Comparisons between two groups were analyzed using unpaired two-tailed Student’s *t* tests and corrected for multiple comparisons using the Holm-Sidak method. Comparisons between more than two groups were analyzed using ordinary one-way analysis of variance (Fig. 6, D and E, and fig. S9, A to C). Survival curves (Figs. 1D and 2D and fig. S3I) were compared by the Log-rank (Mantel-Cox) test. *P* values were calculated with GraphPad Prism software: *P < 0.05; **P < 0.01; ***P < 0.001; ns, not significant.

Sample size determination: No statistical methods were used to predetermine sample size. For in vivo mouse experiments, we always used as many mice per group as possible in an attempt to minimize type I and type II errors and follow ethical guidelines. Experiments were performed at least in two independent measurements as indicated in the figure legends. All sample sizes are indicated in the figure legends.

Blinding: Investigators were not blinded during experiments as we co-caged control and mutant littermates to reduce other variables. Moreover, knockout mice are phenotypically different from their control, preempting any blinding. All animals were re-genotyped after the experimental end point, minimizing any potential bias on data collections. Quantification of images were performed blindly using automated software.

Inclusion/exclusion criteria: No data were excluded.

Replicates: All data describe biological replicates and are indicated in the figure legends.

## Notes

View/request a protocol for this paper from *Bio-protocol*.
