# Supplementary material for: The HUSH complex controls brain architecture and protocadherin fidelity
Source: Sci Adv. 2022 Nov 4;8(44):eabo7247. doi: 10.1126/sciadv.abo7247 (PMC9635835; doi:10.1126/sciadv.abo7247)
Supplement: Supplementary file 1 — Figs. S1 to S9 Table S1 [file sciadv.abo7247_sm.pdf]

Science Advances
AAAS

Supplementary Materials for

The HUSH complex controls brain architecture and protocadherin fidelity

Astrid Hagelkruys *et al.*

Corresponding author: Josef M. Penninger, josef.penninger@ubc.ca;
Astrid Hagelkruys, astrid.hagelkruys@imba.oeaw.ac.at

Sci. Adv. **8**, eabo7247 (2022)
DOI: 10.1126/sciadv.abo7247

This PDF file includes:

Figs. S1 to S9
Table S1

Supplementary Figure 1

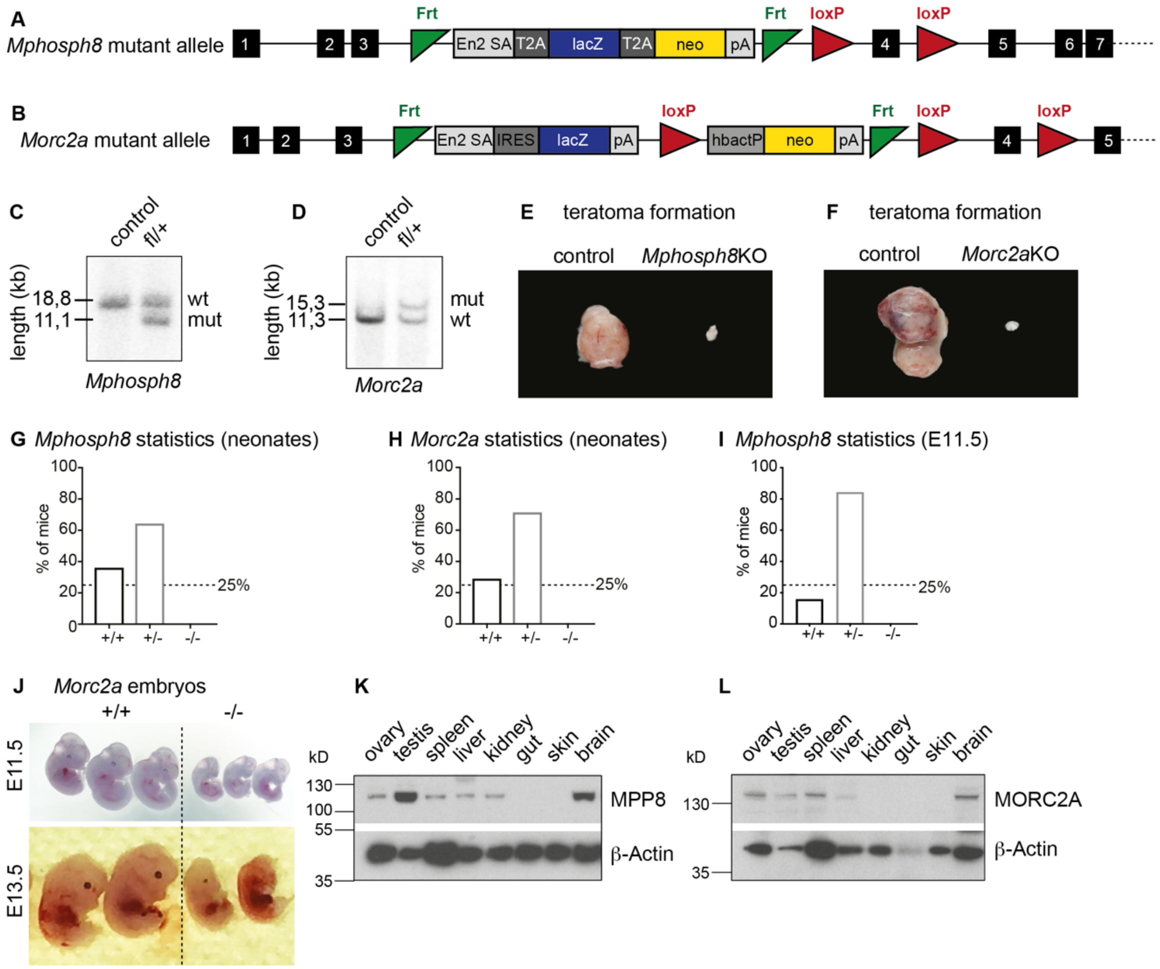

Supplementary Figure 1. Generation of conditional *Mphosph8* or *Morc2a* knock-out ES cells and mice.

(**A,B**) Schematic illustration of the conditional *Mphosph8* (**A**) and *Morc2a* (**B**) alleles. Exons are depicted as black numbered boxes and Frt and loxP sites are shown as green and red triangles, respectively. The LacZ-Neo cassette can be removed by crossing mice to a *FlpE* deleter line. LoxP flanked exon 4 can be removed upon recombination in mice expressing Cre recombinase. En2 SA = mouse En2 splicing acceptor acceptor, T2A = T2A element, pA = polyadenylation signal, IRES = internal ribosome entry side, hbactP = human beta actin promoter.

(**C,D**) Southern blot analysis of control versus heterozygous *Mphosph8*$^{fl/+}$ (**c**) and control versus heterozygous *Morc2a*$^{fl/+}$ (**D**) ES cells indicating correct targeting.

(**E,F**) Teratoma formation 4 weeks after injection of control versus *Mphosph8* knock-out ES cells (**E**) and control versus *Morc2a* knock-out ES cells (**F**).

(**G,H**) Genotype statistics of born mice with *Actin*-Cre-mediated full-body knock-out of *Mphosph8* (**G**, n = 39) and *Morc2a* (**H**, n = 59).

(**I**) Genotype statistics of embryonic day E11.5 mice with *Actin*-Cre-mediated full-body knock-out of *Mphosph8* (n = 19).

(**J**) Representative photographs of control (left) and *Actin*-Cre-mediated full-body knock-out of *Morc2a* (right) embryos at embryonic day E11.5 (upper panel) and E13.5 (lower panel).
(**K,L**) Immunoblot analysis of adult wildtype tissue extracts with antibodies against MPP8 (**K**), MORC2A (**L**) and β-Actin as loading control.

Supplementary Figure 2

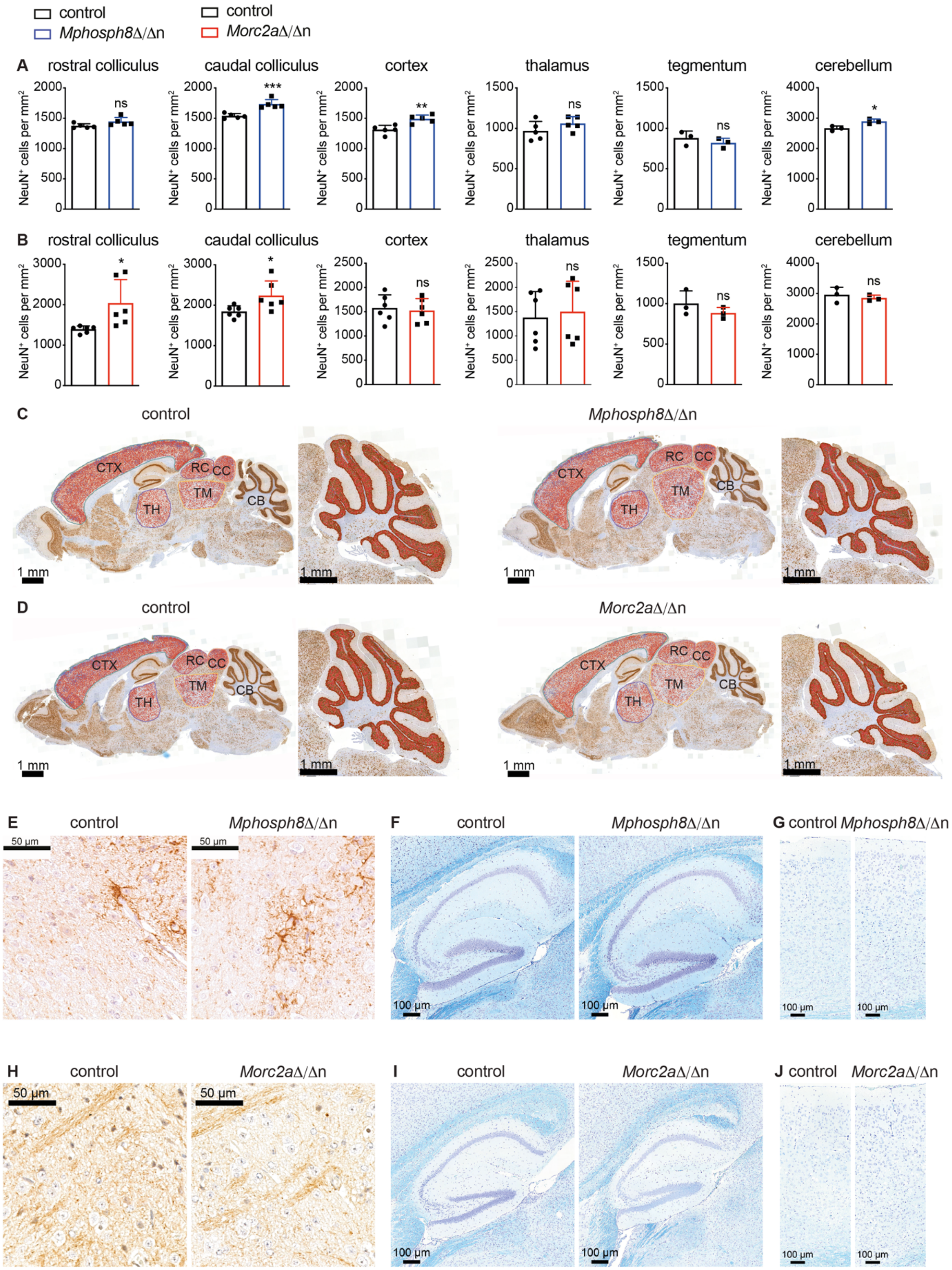

Supplementary Figure 2. Increased density of NeuN+ neurons in defined brain regions of *Mphosph8*- or *Morc2a*-deficient mice.

(**A,B**) Quantification of NeuN positive neurons in six brain regions of *Mphosph8*$^{\Delta/\Delta n}$ (**A**) and *Morc2a*$^{\Delta/\Delta n}$ mice (**B**). For rostral colliculus, caudal colliculus, cerebral cortex and thalamus data from two independent experiments were pooled and shown as mean values +/- SD (n > 4). For the tegmentum and the cerebellum (granular layer) data from one experiment are shown as mean values +/- SD (n = 3).

(**C,D**) Representative immunodetection of NeuN positive neurons in *Mphosph8*$^{\Delta/\Delta n}$ (**C**) and *Morc2a*$^{\Delta/\Delta n}$ (**D**) mice using QuPath v0.2.3 (scale bar = 1 mm). Five brain regions are marked, including the rostral colliculus (RC), caudal colliculus (CC), cerebral cortex (CTX), thalamus (TH) and tegmentum (TM) (left) and the granular layer of the cerebellum (CB) (right).

(**E,H**) Histomorphologic features of the rostral colliculus in *Mphosph8*$^{\Delta/\Delta n}$ (**E**) and *Morc2a*$^{\Delta/\Delta n}$ brains (**H**) stained with Glial Fibrillary Acidic Protein (GFAP).

(**F,I**) Histomorphologic features of the hippocampal formation in *Mphosph8*$^{\Delta/\Delta n}$ (**F**) and *Morc2a*$^{\Delta/\Delta n}$ brains (**I**) stained with Luxol Fast Blue - Cresyl Violet (LFBCV).

(**G,J**) Histomorphologic features of the cerebral cortical layers in *Mphosph8*$^{\Delta/\Delta n}$ (**G**) and *Morc2a*$^{\Delta/\Delta n}$ brains (**J**) stained with Luxol Fast Blue - Cresyl Violet (LFBCV).

For panels A and B each data point represents an individual mouse. p values were calculated using the Student's t test. * $p < 0.05$; ** $p < 0.01$; *** $p < 0.001$; ns, not significant.

Supplementary Figure 3

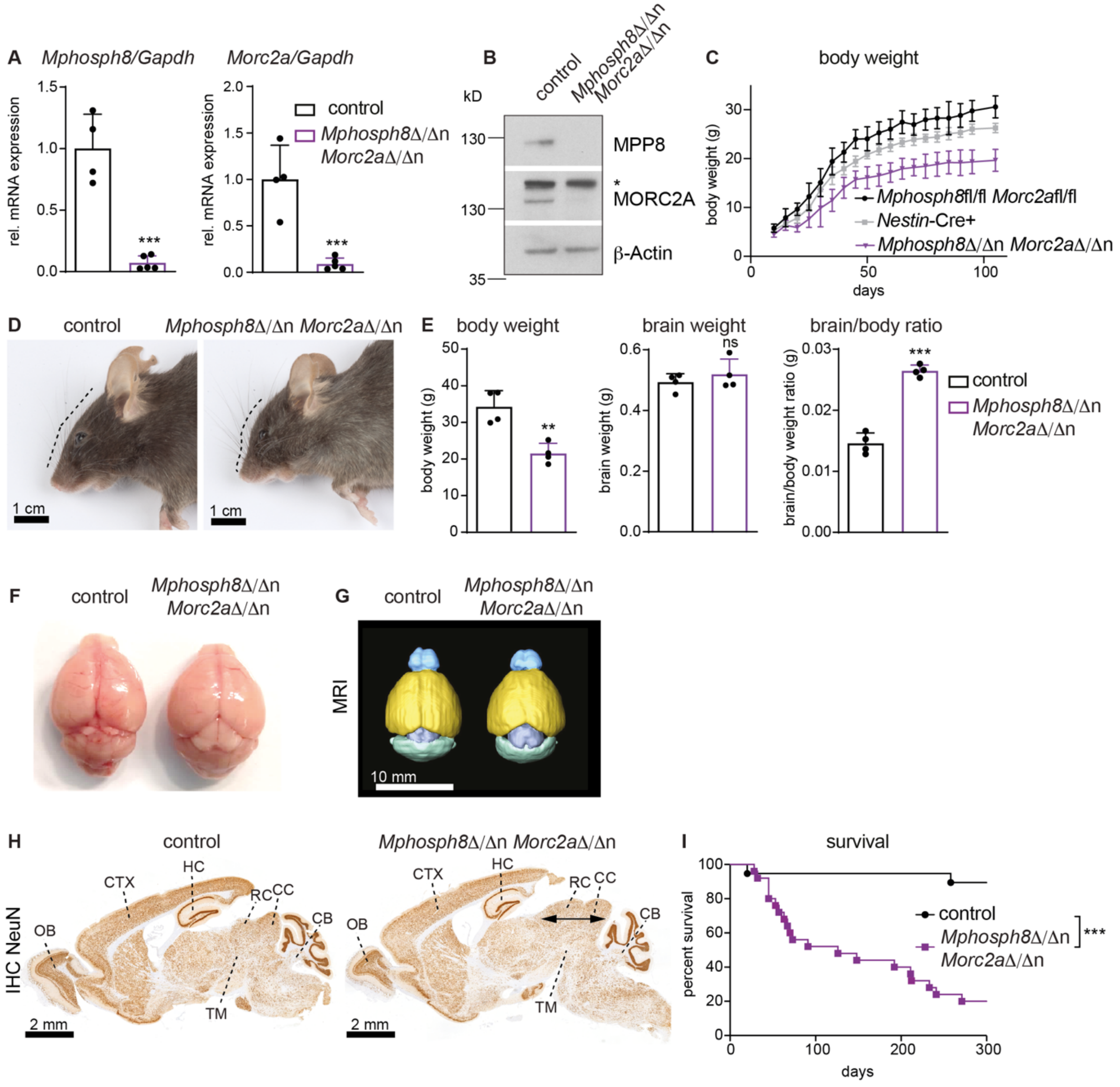

Supplementary Figure 3. ***Mphosph8***$^{\Delta/\Delta n}$ ***Morc2a***$^{\Delta/\Delta n}$ **double knock-out mice.**

(**A**) Relative mRNA expression of *Mphosph8* (left) and *Morc2a* (right) in littermate control and homozygous *Mphosph8*$^{\Delta/\Delta n}$ *Morc2a*$^{\Delta/\Delta n}$ double mutant brains. Mean values +/- SD were normalized to the housekeeping gene *Gapdh* (n > 3). Data are shown for one of two independent experiments.

(**B**) Immunoblot analysis of littermate control versus *Mphosph8*$^{\Delta/\Delta n}$ *Morc2a*$^{\Delta/\Delta n}$ brain extracts with antibodies against MPP8, MORC2A and β-Actin as loading control. The asterisk indicates an unspecific band.

(**C**) Body weights of control *Mphosph8*$^{fl/fl}$ *Morc2a*$^{fl/fl}$ (n = 18), *Nestin*-Cre+ (n = 8) and *Mphosph8*$^{\Delta/\Delta n}$ *Morc2a*$^{\Delta/\Delta n}$ (n = 14) male littermates +/- SD during the first 3 months of age. Data from several litters were pooled.

(**D**) Representative macroscopic photographs of control (left) and *Mphosph8*$^{\Delta/\Delta n}$ *Morc2a*$^{\Delta/\Delta n}$ (right) mouse heads (scale bar = 1 cm).

(**E**) Body weights, brain weights and brain/body ratios of control compared to *Mphosph8*$^{\Delta/\Delta n}$ *Morc2a*$^{\Delta/\Delta n}$ adult (at 3-4 months) littermates. Data from one experiment are shown as mean values +/- SD (n = 4).
(**F**) Representative macroscopic images of a control (left) and a *Mphosph8*$^{\Delta/\Delta n}$ *Morc2a*$^{\Delta/\Delta n}$ (right) littermate adult (at 3-4 months) brain.
(**G**) Representative *in vivo* MRI brain scan of control (left) and *Mphosph8*$^{\Delta/\Delta n}$ *Morc2a*$^{\Delta/\Delta n}$ (right) adult littermates (scale bar = 10 mm). In total 3 mice per genotype were analyzed.
(**H**) NeuN immunohistochemistry (IHC) in adult (at 3-4 months) control (left) and *Mphosph8*$^{\Delta/\Delta n}$ *Morc2a*$^{\Delta/\Delta n}$ (right) brains (scale bar = 2 mm). At least 3 mice per genotype were analyzed by immunohistochemistry. OB = olfactory bulb, CTX = cerebral cortex, HC = hippocampus, RC = rostral colliculus, CC = caudal colliculus, TM = tegmentum, CB = cerebellum. Arrows indicate widening of collicular regions of the midbrain in *Mphosph8*$^{\Delta/\Delta n}$ *Morc2a*$^{\Delta/\Delta n}$ brains.
(**I**) Kaplan-Meier survival curve over the first 300 days of control versus *Mphosph8*$^{\Delta/\Delta n}$ *Morc2a*$^{\Delta/\Delta n}$ littermates. Data from several litters were pooled (n > 18) and survival curves were compared by the Log-rank (Mantel-Cox) test.
For panels A and E each data point represents an individual mouse. p values were calculated using the Student's t test. ** $p < 0.01$; *** $p < 0.001$; ns, not significant.

Supplementary Figure 4

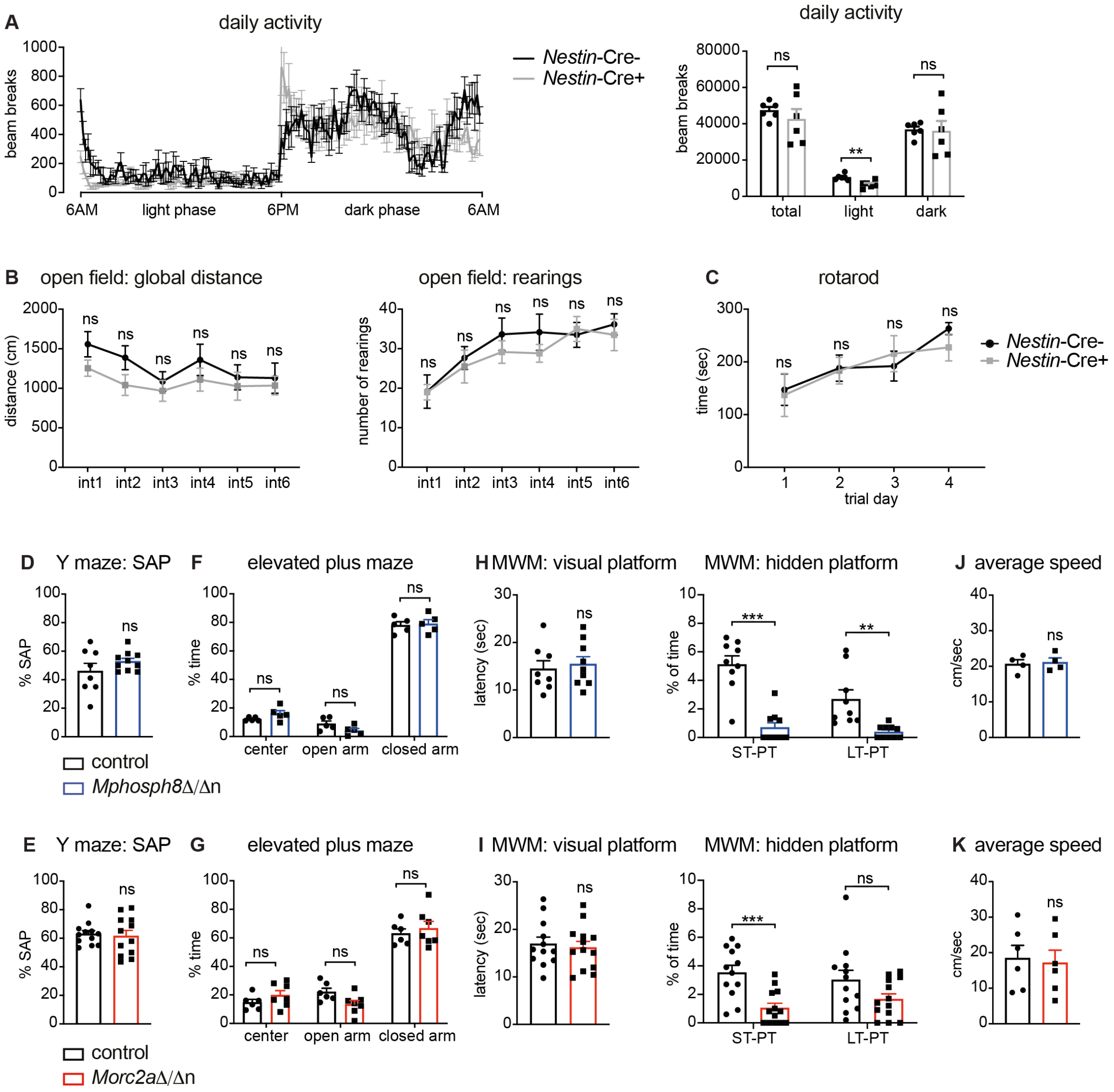

Supplementary Figure 4. Behavioral assessment of *Mphosph8* or *Morc2a* mutant mice.

(**A**) Daily activity in PhenoMaster cages measured by beam breaks of *Nestin*-Cre+ mice compared to *Nestin*-Cre- littermates over 24 hours. Data from two independent experiments were pooled and shown as mean values +/- SEM (n > 5).

(**B**) Global distances travelled and numbers of rearings of *Nestin*-Cre+ mice compared to *Nestin*-Cre- littermates in the open field test. Data from two independent experiments were pooled and shown as mean values +/- SEM (n > 5).

(**C**) Latency to fall from the accelerating Rotarod of *Nestin*-Cre+ mice compared to *Nestin*-Cre- littermates. Data from two independent experiments were pooled and shown as mean values +/- SEM (n > 5).

(**D,E**) Spontaneous alteration performance (SAP) in the Y maze of *Mphosph8*$^{\Delta/\Delta n}$ mice (**D**) and *Morc2a*$^{\Delta/\Delta n}$ mice (**E**) compared to their respective control littermates. Data from two independent experiments were pooled and shown as mean values +/- SEM (n > 7).

(**F,G**) Percent time spent in the three compartments of the elevated plus maze by *Mphosph8*$^{\Delta/\Delta n}$ mice (**F**) and *Morc2a*$^{\Delta/\Delta n}$ mice (**G**) compared to control littermates. Data are representative of one of two independent experiments and shown as mean values +/- SEM (n > 4).
(**H,I**) Latency to find the visual platform in the Morris water maze (left panels) and percent time spent on the hidden platform in the Morris water maze in the short-term (ST-PT) and long-term (LT-PT) probe trial (right) of *Mphosph8*$^{\Delta/\Delta n}$ mice (**I**, n > 7) and *Morc2a*$^{\Delta/\Delta n}$ mice (**I**, n > 11) compared to control littermates. Data from two independent experiments were pooled and shown as mean values +/- SEM.
(**J,K**) Average speed in the Noldus CatWalk system of *Mphosph8*$^{\Delta/\Delta n}$ mice (**J**, n = 4) and *Morc2a*$^{\Delta/\Delta n}$ mice (**K**, n = 6) compared to control littermates. Data are shown as mean values +/- SEM.
For panel A (right part) and panels D-K each data point represents an individual mouse. *p* values were calculated using the Student's *t* test. ** $p < 0.01$; *** $p < 0.001$; ns, not significant.

Supplementary Figure 5

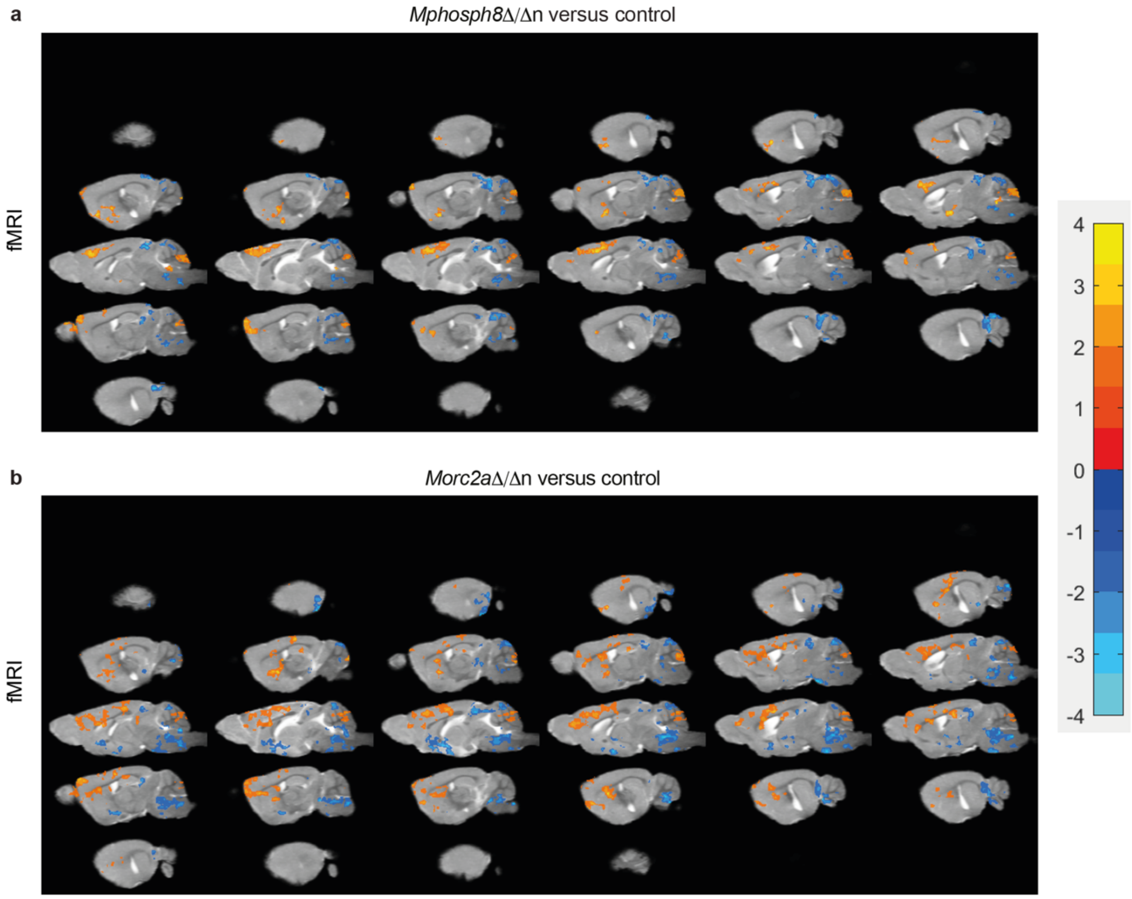

Supplementary Figure 5. Functional Magnetic Resonance Imaging (fMRI) in *Mphosph8* and *Morc2a* knock-out mice.

Resting state fMRI of M*phosph8*$^{\Delta/\Delta n}$ mice (**A**) and *Morc2a*$^{\Delta/\Delta n}$ mice (**B**) compared to control littermates. Per group at least 4 individual mice were scanned. Z-score is shown from -4 (cyan) to 4 (yellow) to show brain regions with significantly lower activation in blue/cyan and higher activation in orange/yellow compared to the control group.

Supplementary Figure 6

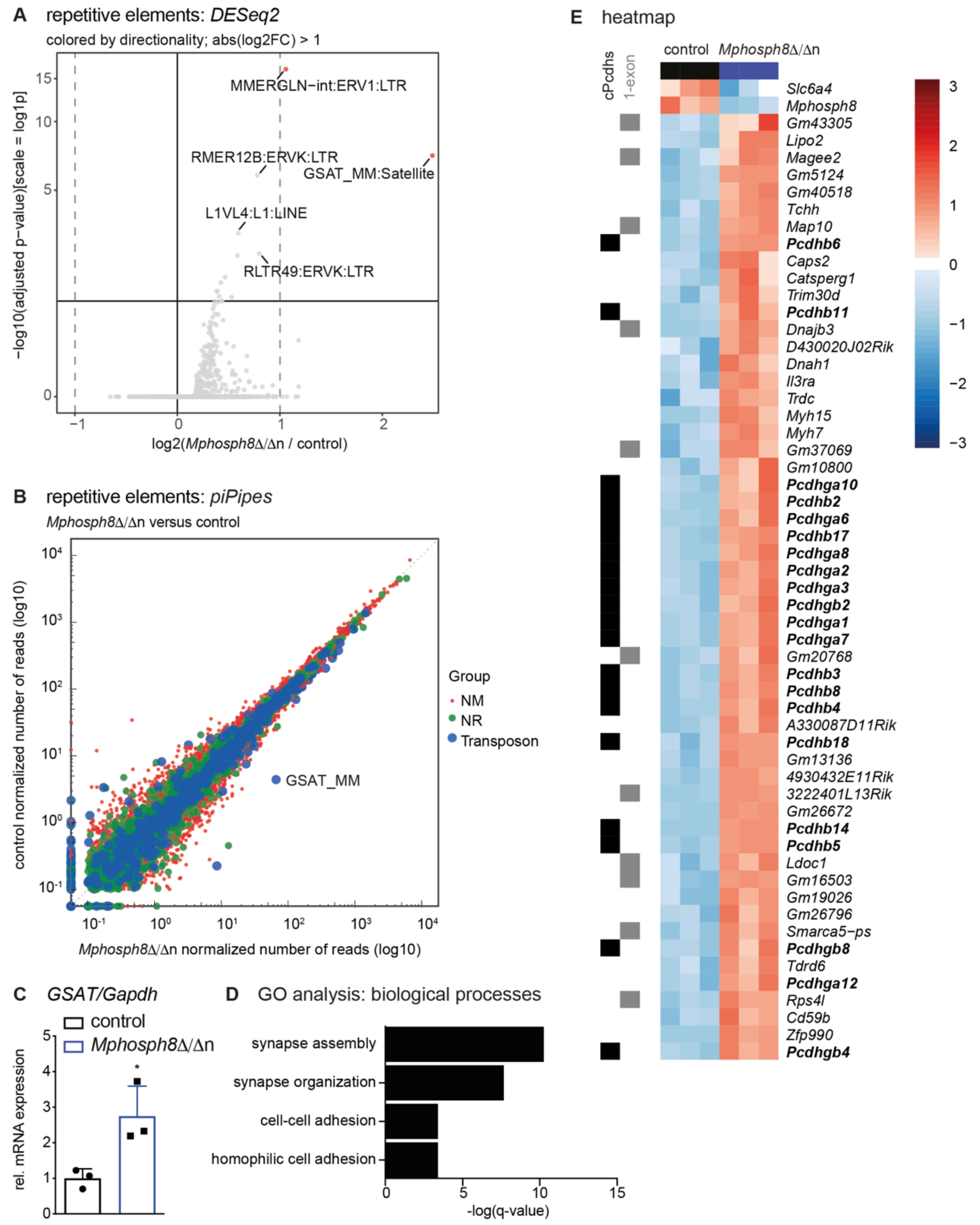

Supplementary Figure 6. Upregulation of major satellite repeats and the protocadherin cluster in the brains of *Mphosph8* knock-out mice.

(**A**) Volcano plot of differential expression analysis (*DESeq2*) to estimate repetitive element enrichment in an RNA-seq experiment of 3 littermate control and 3 *Mphosph8*$^{\Delta/\Delta n}$ brains on postnatal day 14. Elements with a fold change > 2 (log2 fold change > 1) are shown in red, others in gray. GSAT_MM = major satellite repeats, MMERGLN-int:ERV1:LTR = LTR retrotransposon belonging to the ERV1 family.

(**B**) *piPipes* analysis to estimate repetitive element enrichment in an RNA-seq experiment of 3 littermate control and 3 *Mphosph8*$^{\Delta/\Delta n}$ brains on postnatal day 14. NM = mRNA, NR = ncRNA. GSAT_MM = major satellite repeats.
(**C**) Relative mRNA expression of major satellites repeats (GSAT) in littermate control and *Mphosph8*$^{\Delta/\Delta n}$ brains. Mean values +/- SD were normalized to the housekeeping gene *Gapdh* (n = 3).
(**D**) Biological gene ontology (GO) processes overrepresented among the gene set of 55 upregulated genes in an RNA-seq experiment of 3 littermate control and 3 *Mphosph8*$^{\Delta/\Delta n}$ brains on postnatal day 14 (adjusted p value < 0.05). The negative log10 of the q-value to control the positive false discovery rate for multiple testing is shown.
(**E**) Heatmap of differentially regulated genes (adjusted p value < 0.05, fold change >2) found in the RNA-seq experiment illustrated in **(D)** showing approximately half of the upregulated genes are from the protocadherin gene cluster (cPcdh, marked in black) and 11 more upregulated genes are also intronless (1-exon, grey). Z-score is shown from -3 (blue) to 3 (red).
(**F**) Relative mRNA expression of *Mphosph8*, *Morc2a* and *Pcdhb14* in littermate control, *Mphosph8*$^{\Delta/\Delta n}$ and *Morc2a*$^{\Delta/\Delta n}$ sorted brain NeuN+ nuclei. Mean values +/- SD were normalized to the housekeeping gene *Gapdh* (n = 2).
For panel C each data point represents an individual mouse. p values were calculated using the Student's t test. * $p < 0.05$.

Supplementary Figure 7

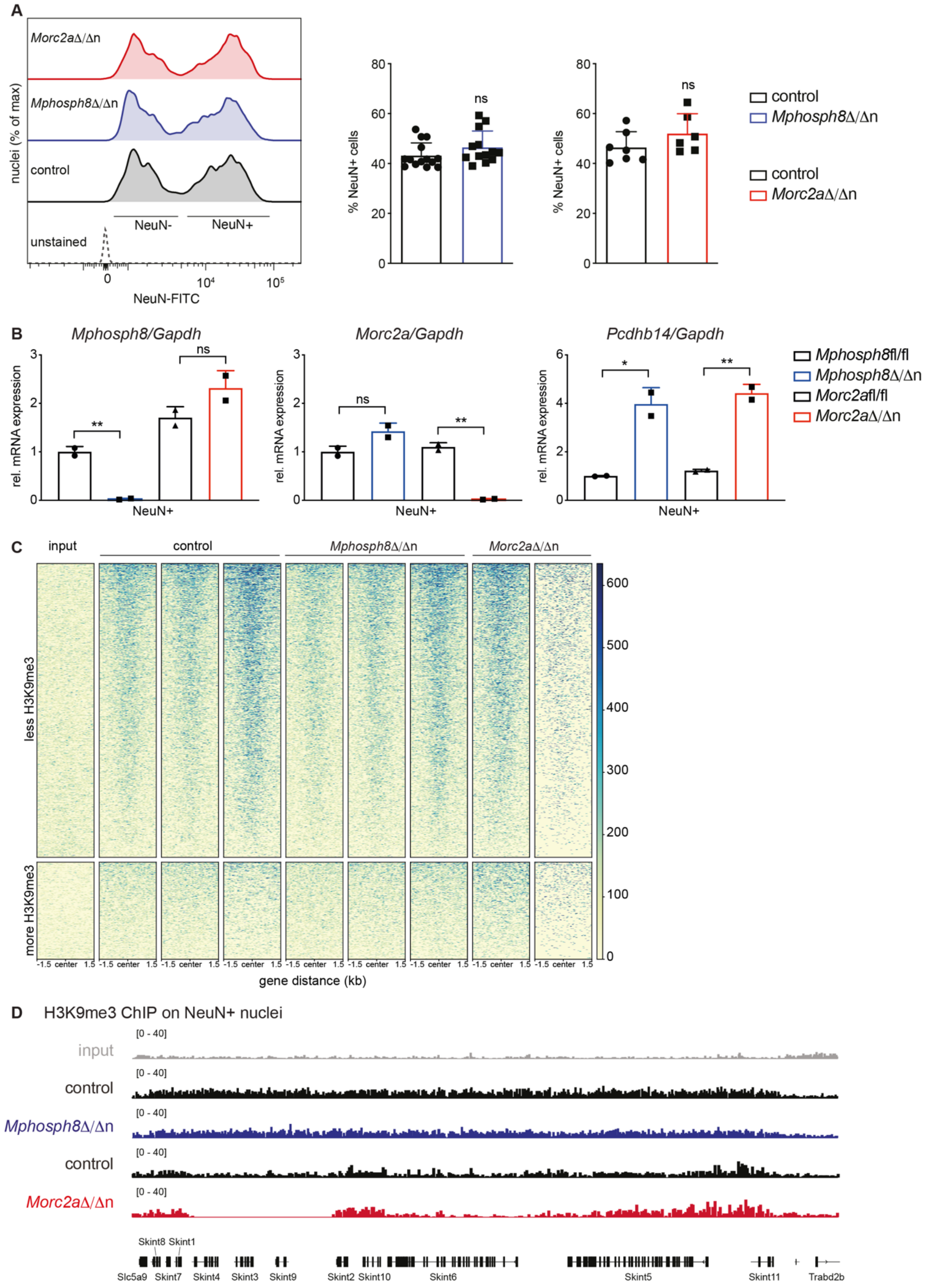

Supplementary Figure 7. Analysis of NeuN positive nuclei in *Mphosph8* and *Morc2a* knock-out mice.

(**A**) Representative plots (left) and quantifications (right) of flow cytometry-based sorting of NeuN-immunotagged brain nuclei in control versus *Mphosph8*$^{\Delta/\Delta n}$ (n > 12) and control versus *Morc2a*$^{\Delta/\Delta n}$ (n > 5) mice. The gray dotted line in the histogram indicates unstained nuclei as background control. For the graphs on the right, data from several independent experiments were pooled and shown as mean values +/- SD.

(**B**) Relative mRNA expression of *Mphosph8*, *Morc2a* and *Pcdhb14* in littermate control, *Mphosph8*$^{\Delta/\Delta n}$ and *Morc2a*$^{\Delta/\Delta n}$ sorted brain NeuN$^+$ nuclei. Mean values +/- SD were normalized to the housekeeping gene *Gapdh* (n = 2).

(**C**) Heatmap of normalized ChIP-seq signals of the NeuN-sorted control, *Mphosph8*$^{\Delta/\Delta n}$ and *Morc2a*$^{\Delta/\Delta n}$ replicates in 3kb windows around the center of the differentially H3K9me3 methylated regions reported in *Setdb1* knock-out versus wildtype cortical neurons (Supplementary Table 1 of (*14*)). Signal intensities from 0 (yellow) to 600 (blue) are shown.

(**D**) Normalized read density plots of H3K9me3 ChIP-seq in brain NeuN$^+$ nuclei from control, *Mphosph8*$^{\Delta/\Delta n}$ and *Morc2a*$^{\Delta/\Delta n}$ mice for the *Skint* gene cluster on chromosome 4.

For panel A and B each data point represents nuclear preparations originating from an individual mouse. *p* values were calculated using the Student's *t* test. * $p < 0.05$; ** $p < 0.01$; ns, not significant.

Supplementary Figure 8

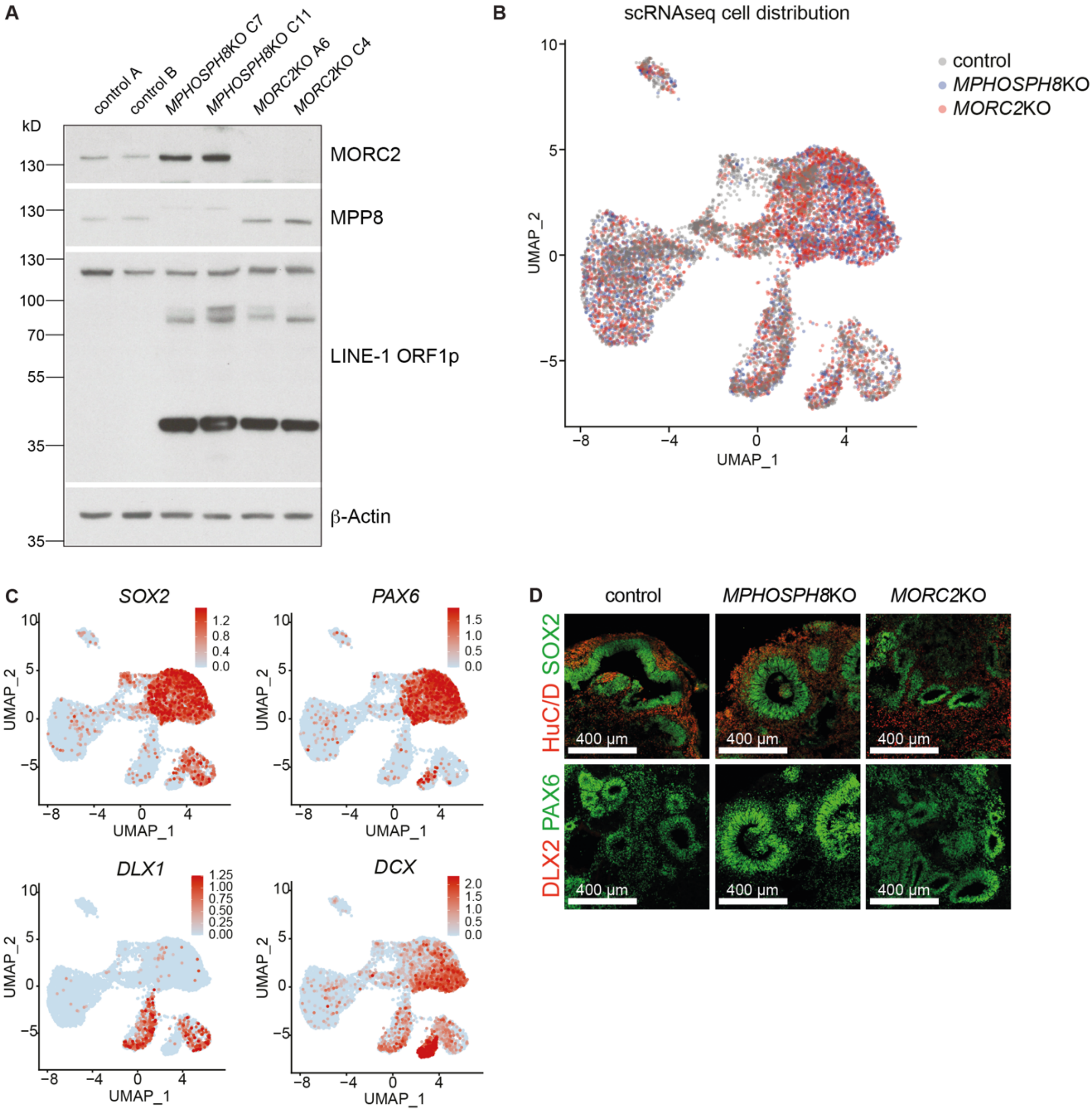

Supplementary Figure 8. Characterization of *MPHOSPH8KO* and *MORC2*KO human embryonic stem cells and human cerebral organoids.

(**A**) Immunoblot analysis of two independent clones of control, *MPHOSPH8*KO and *MORC2*KO H9 human embryonic stem cells used for cerebral organoid generation. The blots were incubated with antibodies against MPP8, MORC2A, LINE-1 ORF1p and β-Actin as loading control.

(**B**) Uniform manifold approximation and projection (UMAP) plot of single-cell RNA-sequencing from day 27 cerebral organoids. Approximately 3400 cells per group were analyzed and are color-coded by genotype. For each group two organoids (each organoid originating from an independent ESC clone) were pooled.

(**C**) Individual UMAP plots for the neuronal precursor marker genes *SOX2*, *PAX6*, *DLX1*, and also *DCX* as marker for immature neurons for all cells shown in (**B**).

(**D**) Representative staining of neural progenitors (SOX2, green) and neurons (HuC/D, red) (upper panels) as well as dorsal neural progenitors (PAX6, green) and the ventral transcription factor (DLX2, red) (lower panels) of day 60 control, *MPHOSPH8*KO and *MORC2*KO cerebral organoids (scale bar = 400 µm).

Supplementary Figure 9

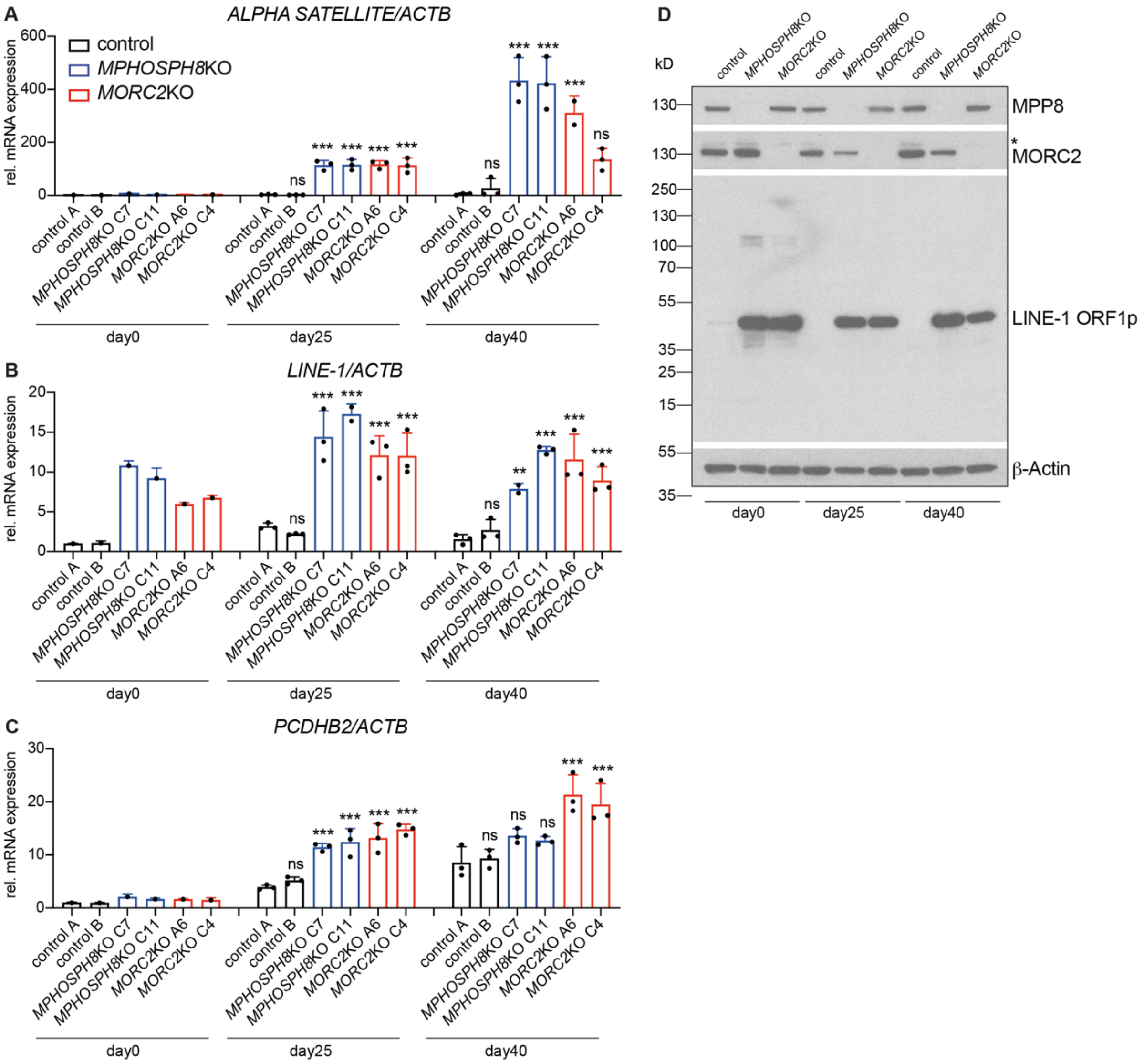

Supplementary Figure 9. *MPHOSPH8*KO or *MORC2*KO human cerebral organoids show a strong upregulation of repetitive elements and clustered protocadherins.

(**A,B,C**) Relative mRNA expression of alpha satellites (**A**), LINE-1 transposable elements (**B**) and *PCDHB2* (**C**) in two independent clones of control, *MPHOSPH8*KO and *MORC2*KO hESCs (day 0) and cerebral organoids (day 25 and day 40). Mean values +/- SD were normalized to the housekeeping gene β-Actin (*ACTB)* (n = 1 for day 0 ESC, n = 3 for day 25 and day 40 cerebral organoids). Data are shown for one of three independent experiments.

(**D**) Representative immunoblot of control, *MPHOSPH8*KO and *MORC2*KO H9 hESCs (day 0) and cerebral organoids (day 25 and day 40). The blot was incubated with antibodies against MPP8, MORC2A, LINE-1 ORF1p and β-Actin as loading control. The asterisk indicates an unspecific band.

For panels A-C each data point represents an individual organoid and p values were calculated using one-way ANOVA and Dunnett's multiple comparisons test compared to control A for each time point. ** $p < 0.01$; *** $p < 0.001$; ns, not significant.

Supplementary Table 1.

Genotyping primers:	
Mphosph8	GTTCTTTATGAGAGCACCAAG
	TCTACACACGCCTGAGC
	WT allele: 467 bp, floxed allele: 584 bp
Morc2a	TTCACATGCCCAGGTACATTCAC
	TTCTGGTAGCATCAGCATCTCTG
	WT allele: 581 bp, floxed allele: 778 bp
Cre	TAATCGCCATCTTCCAGCAG
	CAATTTACTGACCGTACAC
	Cre: 1000 bp
FlpE	GTGGATCGATCCTACCCCTTGCG
	GGTCCAACTGCAGCCCAAGCTTCC
	FlpE: 700 bp
Thy1-Egfp	ACAGACACACACCCAGGACA
	CGGTGGTGCAGATGAACTT
	CTAGGCCACAGAATTGAAAGATCT
	GTAGGTGGAAATTCTAGCATCATCC
	Internal positive control: 324 bp, EGFP transgene: 415 bp
Primers for mouse cDNA:	
Gapdh	GTCGGTGTGAACGGATTTGG
	GACTCCACGACATACTCAGC
Mphosph8	GCTACCATCACCTGTGTTTG
	TCTTCTTTGTCCAAATCATACC
Morc2a	GCTGATGCTACCAGAATAGAC
	GGCTGACTTCCCAAACTG
GSAT	GATTTCGTCATTTTTCAAGTCGTC
	GCACACTGAAGGACCTGGAATATG
Pcdhb3	CAGGTCCACATCCTGGTCTT
	TCCCGTATCCAAGTCAGAGG
Pcdhb14	GCTGTCATTCCAACTGCTCA
	GGCAGAGTGTGGAGGTTCTC
Primers for mouse ChIP:	
Pcdhb14+69	CTGCGTCCAGCAACATTAAA
	AGATAATGGCGGTCACTTGC
Primers for human cDNA:	
ACTB	TTGCCGACAGGATGCAGAAGGA
	AGGTGGACAGCGAGGCCAGGAT
MPHOSPH8	CGATCACAAAACCAAGGAAAA
	TTCTCATCTGCTTGGCACAC
MORC2	TGGCTTTCACAAATTACAGCA
	TT GCATTATCAACCAGTTCAGC
LINE-1	TAACCAATACAGAGAAGTGC
	GATAATATCCTGCAGAGTGT
alpha satellites	AAGGTCAATGGCAGAAAAGAA

	CAACGAAGGCCACAAGATGTC
PCDHB2	GGGGCTGGAGATAGGAGAAC
	TAACAACAAATCCCCGGTCT
PCDHB5	TGGCTCTGAGGCAGTTAGGT
	TAGAAGCAAATTGCCGGTCT
PCDHB14	CCTTGTTTTGCTGGGATTGT
	CCCTAGGTCCCTCGCTAGAT

<u>Antibodies for immunoblots and ChIP:</u>

β-Actin	A5316 (Sigma)
MPP8	16796-1-AP (Proteintech)
MORC2	PA5-51172 (ThermoFisher)
GAPDH-HRP	3683 (Cell Signaling)
NeuN	MAB377 (Merck Millipore)
LINE-1 ORF1p	MABC1152 (Merck Milipore)
C-terminal H3	clone 1B1-B2 (Active Motif)
H3K9me3	ab8898 (Abcam)

<u>Primary and secondary antibodies for immuohistochemistry:</u>

SOX2	AB5603 (Merck), rabbit
PAX6	ab195045 (Abcam), rabbit
DLX2	sc-393879 (Santa Cruz), mouse
HuC/D	A-21272 (Invitrogen), mouse
anti-rabbit IgG, Alexa Fluor 555	A-21428 (Invitrogen)
anti-mouse IgG, Alexa Fluor 633	A-21053 (Invitrogen)
